# H4-methylation regulators mediated epitranscriptome patterns and tumor microenvironment infiltration characterization in hepatocellular carcinoma

**DOI:** 10.1186/s13148-023-01460-6

**Published:** 2023-03-17

**Authors:** Linyuan Yu, Tao Ji, Wei Liao, Yuyan Xu, Yinghao Fang, Qing Zhu, Jianmin Nie, Dinghua Yang

**Affiliations:** 1 Unit of Hepatobiliary Surgery, Department of General Surgery, Nanfang Hospital, Southern Medical University, Guangzhou, 510515 Guangdong Province China; 2 General Surgery Center, Department of Hepatobiliary Surgery II, Guangdong Provincial Research Center for Artificial Organ and Tissue Engineering, Guangzhou Clinical Research and Transformation Center for Artificial Liver, Institute of Regenerative Medicine, Zhujiang Hospital, Southern Medical University, Guangzhou, Guangdong Province China

**Keywords:** Histone H4 methylation, Hepatocellular carcinoma, Molecular subtype, Tumor microenvironment, Immunotherapy biomarker

## Abstract

Epigenetic modifications are involved in the remodeling of the tumor microenvironment (TME) and the regulation of immune response. Nonetheless, the role of histone H4 methylation (H4M) modification in the TME and immune regulation of hepatocellular carcinoma (HCC) is unknown. As a result, the purpose of this research is to discover H4M-mediated modification patterns and their effects on TME and immunologic characteristics in HCC. A total of 2305 samples were enrolled from 13 different cohorts. With the help of consensus clustering analysis, three distinct H4M modification patterns were identified. The cell-infiltrating characteristics of TME under these three patterns were highly consistent with their enriched biological processes and clinical outcome. The H4Mscore was then created using principal component analysis algorithm to quantify the H4M modification pattern of each individual tumor and was systematically correlated with representative tumor characteristics. We found that analyzing H4M modification patterns within individual tumors could predict TME infiltration, homologous recombination deficiency (HRD), intratumor heterogeneity, proliferation activity, mRNA stemness index, and prognosis. The group with a low H4Mscore had an inflamed TME phenotype and a better immunotherapy response, as well as a better survival outcome. The prognostic value of H4Mscore was validated in three internal cohorts and five external cohorts, respectively. In external immunotherapy cohorts, the low H4Mscore was also linked to an enhanced response to anti-PD-1/L1 and anti-CTLA4 immunotherapy and a better prognosis. This study revealed that H4M modification played an important role in forming TME diversity and complexity. Evaluating the H4M modification pattern of individual tumors could help us learn more about TME and develop more effective immunotherapy strategies.

**Supplementary Information:**

The online version contains supplementary material available at 10.1186/s13148-023-01460-6.

## Introduction

Hepatocellular carcinoma (HCC) is a global problem that endangers human health due to the extremely high number of new and fatal cases [1]. The heterogeneity of HCC has received increased attention in recent years, placing it at the forefront of all solid tumors [2]. Because of heterogeneity, HCC can be classified into different molecular subtypes with distinct biological characteristics and treatment responses [3, 4]. Surgical removal is the backbone treatment for patients with HCC, but most patients have lost the opportunity to accept an operation when they are attender. Unfortunately, the prognosis of HCC is very poor, with only 18% of patients surviving five years after diagnosis [5].

As a complex ecosystem comprised of cancer cells, stromal cells, infiltrated immune cells and extracellular matrix, the tumor microenvironment (TME) has been widely acknowledged to play a pivotal role in carcinogenesis, metastatic dissemination, and anti-tumor immunotherapy response [6–8]. Due to the innate anti-inflammatory immune contexture for tolerance to intestinal antigens and predisposition induction of cancer cells, the TME of HCC will always be perceived as suppressive [9, 10]. Cancer cells and multiple extracellular components interplay in direct or indirect ways to elicit special biological changes in epigenetic features, thereby leading to tumor progression and immune evasion [11–13]. Immune checkpoint blockers have so far been effective for only a fairly small percentage of patients [14, 15]. Therefore, it is urgent to discover more effective biomarkers for immunotherapy.

Epigenetic modifications have exhibited robust effects on tumor progression and anti-tumor immune response [16–18]. As a core member of histone modification, histone H4 methylation (H4M) is dynamic and reversible to some extent, which can be regulated by multiple methyltransferases, demethylases, and binding proteins and is closely related to multiple cancer phenotypes, covering cancer cell proliferation, migration, and drug resistance [19–21]. The dysregulation of H4M modification is a meaningful signature for multiple malignancies [22–25]. For the cancer genome or specific oncogenes, increasing evidence confirms that the reduction of H4M modification is closely associated with oncogene activation and causes adverse outcomes in breast, colon, bladder, ovarian, and hematological cancers [26–28]. However, the role of H4M modification in the carcinogenesis and progression of HCC has received scant attention.

Previous studies have confirmed the vital role of H4M modification in cancer progression and its capability as a therapeutic target. However, the specific coordinated effects of H4M regulators in HCC need to be further explored. Therefore, a multi-omics analysis was performed to identify H4M-related modification patterns and their effects on TME and immune regulation in HCC. Three distinct H4M modification patterns were identified, and as a result, a significant difference was analyzed in TME and immunological characteristics. Furthermore, an H4M-related biomarker was created to further quantify the degree of individual H4M modification and reveal the pivotal role of H4M modification in HCC. This study highlighted the specific changes in TME and immunological characteristics induced by H4M modification and characterized a novel signature for HCC risk prediction.

## Methods

### Hepatocellular carcinoma data acquisition and preprocessing

**Fig. 1:**
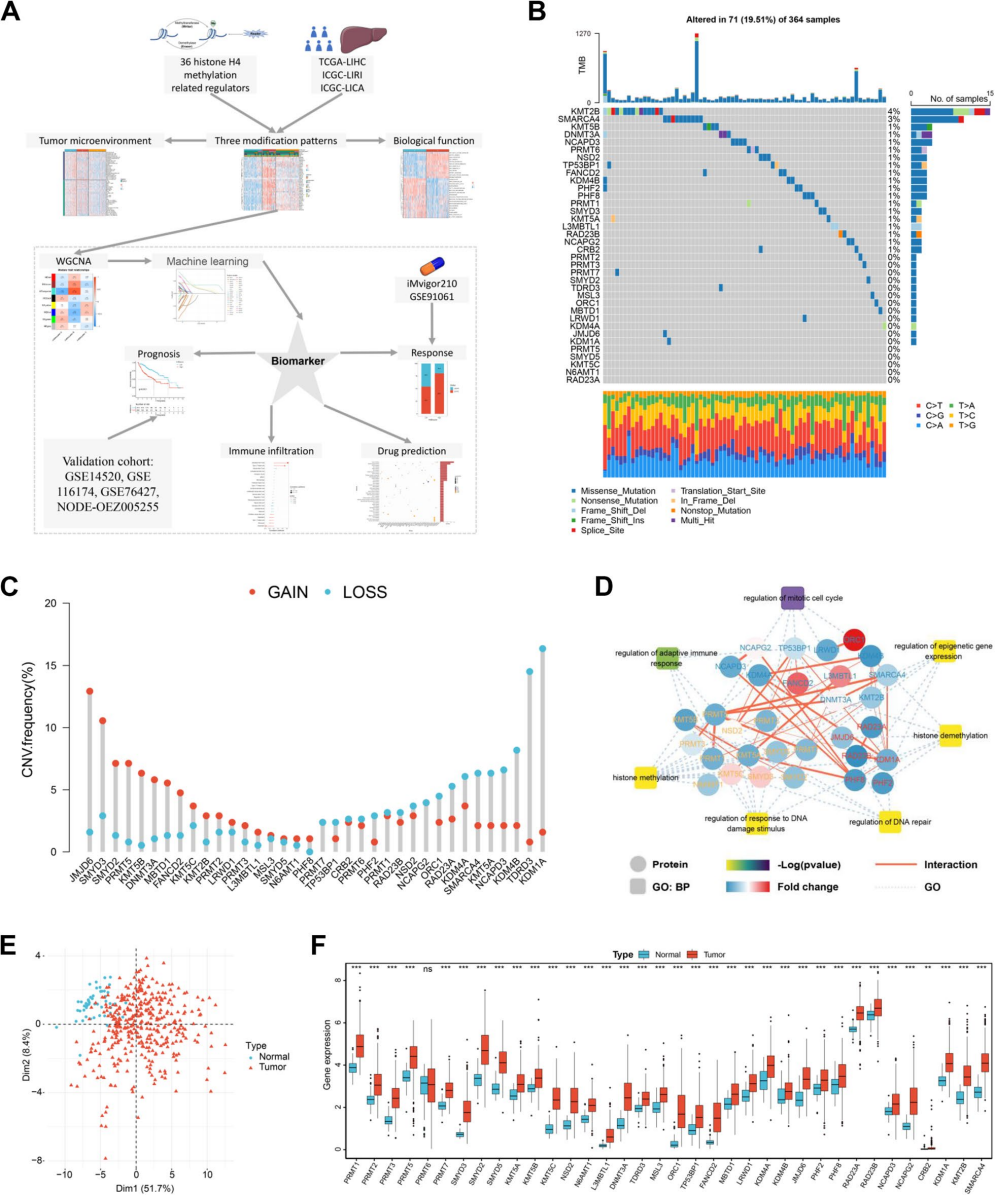
Expression and genomic variation landscape of 36 H4 methylation (H4M) regulators in hepatocellular carcinoma (HCC). **A** The main workflow of this study. **B** The mutation frequency of 36 H4M regulators in 371 patients with hepatocellular carcinoma from the TCGA-LIHC cohort. **C** Copy number variation (CNV) frequency of H4M regulators in TCGA-LIHC. **D** The interaction diagram of proteins involved in histone methylation, histone demethylation, regulation of response to DNA damage stimulus, regulation of epigenetic gene expression, regulation of DNA repair, regulation of mitotic cell cycle, and regulation of adaptive immune response. Red solid lines represent protein–protein interactions; pale blue dotted lines represent GO processes. The color bar from red to blue depicts the fold change of protein levels from increasing to decreasing. The significance of the pathways represented by − log (*p* value) was shown by color scales with dark purple as the most significant. **E** Principal component analysis of the 36 H4M regulators in normal and HCC patients (blue indicates normal and red indicates HCC patients). **F** Expression of 36 H4M regulators between normal and HCC patients. *, **, and *** mean *p* < 0.05, < 0.01, and < 0.001, respectively

This study is summarized by a flowchart, as depicted in Fig. 1A. HCC and adjacent liver tissues were obtained from patients who had undergone radical resection between November 2010 and November 2020 at Nanfang Hospital, Southern Medical University. A total of 30 patients were enrolled in this study. Samples were obtained with the consent of the patients and the hospital ethics committee (approval document number: NFEC‐2018‐004). The human HCC cell line Huh‐7 was obtained from the Chinese Academy of Sciences’ Type Culture Collection. Cells were cultured with DMEM (C11995500BT; Gibco) with 10% FBS (A3160801; Gibco) and incubated at 37 °C in a humidified atmosphere with 5% CO_2_.

Additionally, publicly available data were converged from the Cancer Genome Atlas (TCGA, https://portal.gdc.cancer.gov/) and International Cancer Genome Consortium (ICGC, https://dcc.icgc.org/) databases. Eight HCC cohorts (GPL3921-GSE14520, GPL571-GSE14520, GSE109211, GSE 116174, GSE54236, GSE63898, GSE76427, NODE-OEZ005255) from the Gene Expression Omnibus (GEO, https://www.ncbi.nlm.nih.gov/gds) and the National Omics Data Encyclopedia (NODE, https://www.biosino.org/node/index) were employed as validation cohorts. Two immunotherapeutic cohorts were brought into this study to explore the value of the H4M scoring system in predicting response to immune checkpoint blockers. The IMvigor210 cohort, an open anti-PD-L1 immunotherapy cohort, included 310 patients with advanced urothelial carcinoma treated with atezolizumab [29]. The GSE91061 cohort included 65 patients with melanoma treated with anti-PD1 and anti-CTLA4 antibodies [30]. These two immunotherapy cohorts were incorporated into this study. The RNA sequencing data were accurately converted to transcripts per kilobase million format. Batch effects were reduced by the "ComBat" algorithm using the "sva" R package [31]. All baseline information on the available data is summarized in Additional file 14: Table S1.

### Unsupervised clustering for 36 H4M regulators

Multi-studies yielded 36 genes as H4M-initial biomarkers, including 14 writers, 6 erasers, and 16 readers **(**Additional file 14: Table S2). Based on the expression of 36 H4M regulators, an unsupervised clustering algorithm was used to identify unique H4M modification patterns and classify patients. The "ConsensusClusterPlus" package was applied to execute this consistent clustering with 1,000 repetitions to guarantee the stability of classification [32]. Then, the reliability of this classification was confirmed by Kaplan–Meier survival curves and principal component analysis.

### Implementation of gene set variation analysis (GSVA)

In order to determine the difference in biological processes among distinct H4M modification patterns, a GSVA analysis was implemented. By mapping our gene-expression data into the gene set "h.all.v7.5.1.symbols" from the Molecular Signatures Database (MSigDB, http://www.gsea-msigdb.org/gsea/msigdb/index.jsp), relative enrichment score was calculated using the "GSVA" R package [33, 34].

### Estimation of HCC TME cell infiltration

Single sample gene set enrichment analysis was applied to quantify the abundance of immune cells in the TME of HCC [35]. All HCC samples could obtain an enrichment fraction for each immune-infiltrating cell according to their gene-expression map. A total of 23 immune-infiltrating cells were identified, including T cells, B cells, neutrophils, macrophages, dendritic cells, and so on. The markers used to characterize 23 immune-infiltrating cells were originated from the research by Charoentong [36].

### Identification of hub genes and functional annotation

First, the "limma" R package was utilized to identify DEGs between different H4M modification patterns according to the cutoff criteria: *p* value < 0.001 and |logFC|≥ 1. Then, weighted gene co-expression network analysis (WGCNA) was conducted to determine gene modules connected with distinct H4M modification patterns using the R package “WGCNA”. Additionally, a scatterplot of gene significance versus module membership in particular modules was plotted to further obtain hub genes.

The R package "clusterProfiler" was used to conduct Gene Ontology (GO) functional enrichment analysis for 36 H4M regulators and Kyoto Encyclopedia of Genes and Genomes (KEGG) functional enrichment analysis. The "CBNplot" R package was utilized to explore pathways or gene regulatory relationships.

### Machine learning and the generation of H4Mscore signature

In order to quantify the H4M modification pattern of each individual tumor, a scoring system was developed. The H4M-related scoring system, which we termed the H4Mscore. Certain steps were taken to establish the H4M signature. At first, a consensus clustering algorithm was used to define the number of gene clusters using the hub genes discovered by WGCNA analysis. Then, univariate Cox (uniCox) regression analysis was performed for these hub genes. The genes with significant prognostic value were selected for further analysis. Subsequently, overfitting genes were minimized by the Least Absolute Shrinkage and Selection Operator (LASSO) regression algorithm. Finally, principal component analysis (PCA) was applied for modeling. Both principal components 1 and 2 were chosen to act as signature scores. Then, the H4Mscore was defined using the formula [37, 38]: H4Mscore = ∑ (PC1i + PC2i), where i is the expression of H4M-related feature genes.

### RT‐qPCR

Total RNA from clinical samples and HCC cell line was isolated using TRIzol reagent (15596018; Thermo Fisher Scientific). Then, the extracted RNA was reverse‐transcribed to cDNA using an Evo M‐MLV RT kit (AG11711; Accurate Biology) according to the manufacturer’s instructions. Next, cDNA was quantified by real‐time PCR using SybrGreen qPCRmasterMix (4309155; Thermo Fisher Scientific) on a StepOnePlus real‐time PCR system (Applied Biosystems). The 18S rRNA was chosen as the reference gene, and the 2^−ΔΔCt^ formula was used to calculate the expression of the target genes. The primer sequences are summarized in Additional file 14: Table S3.

### Cell transfections, CCK8 assays, and transwell assays

All siRNA oligonucleotides were synthesized by RiboBio, and the siRNA duplex sequences are presented in Additional file 14: Table S4. After 24 h of culture in 12-well plates, siRNAs were transfected into the cells. Following the manufacturer's instructions, all transfections were carried out using jetPRIME reagent (114–15; Polyplus). Cell viability was assessed by the CCK‐8 (CK04; Dojindo Laboratories) assay. The cells (500 cells/well) were seeded in 96‐well plates. Cell viability was measured after adhesion at 24, 48, 72, and 96 h. For Transwell assays, cells were detached and suspended in medium with 10% FBS and seeded (5 × 10^4^/well) into the upper chamber. The bottom chamber was filled with 20% FBS. After incubation for 48 h, cells that migrated to the lower filter surfaces were fixed with polyformaldehyde for 20 min, stained with 0.1% crystal violet for 20 min, and digitally imaged under a high magnification of microscope (100×).

### Immunotherapy response prediction

From the TIDE database (http://tide.dfci.harvard.edu/), the predicted immune response information, TIDE score, microsatellite instability (MSI) score, immune exclusion score, and immune dysfunction score of HCC patients were obtained. Likewise, immunophenoscore was retrieved from the Cancer Immunome Atlas (TCIA, https://tcia.at/home).

### Statistical analysis

Wilcoxon tests were used to compare differences between two groups, while Kruskal-Wallis tests were utilized to compare differences between three or more groups. T test was used to compare paired samples. Correlation coefficients and p values were conducted by Spearman and Pearson correlation analyses. The "surv-cutpoint" function of the "survminer" R package was used to determine the optimal cutoff value for separating HCC patients into high and low H4Mscore groups. One-class logistic regression algorithm was utilized to calculate the stemness index [39]. All statistical p values were two-sided, with *p* < 0.05 as statistically significant. All data processing was carried out using R software (version 4.1.3).

## Results

### Landscape of genetic variation of H4M regulators in HCC

Among 364 HCC samples, genetic alterations in 31 H4M regulators were detected in 71 samples, with a lower frequency of 19.51%. The forms of somatic mutation were multitudinous. Missense mutation was the most common type of mutation, followed by frameshift deletion (Fig. 1B). Further analyses revealed conspicuous mutation co-occurrence relationships involving TDRD3, DNMT3A, LRWD1, PHF2, PHF8, PRMT1, PRMT6, PRMT7, CRB2, KDM4B, KDM1A, JMJD6, FANCD2, and KMT5B (Additional file 1: Fig. S1A). Moreover, widespread CNV alterations were found in 36 H4M regulators (Fig. 1C). The chromosome locations of 36 H4M regulators are present in Additional file 1: Fig. S1B. Then, universally positive correlations between CNV and mRNA expression levels of H4M regulators were discovered (Additional file 1: Fig. S1C). DNA methylation on gene regulatory sequences was known to inhibit gene transcription, whereas low levels of DNA methylation led to transcriptional activation. Therefore, the negative correlation between DNA methylation levels and mRNA expression was common. This phenomenon could be found in most of the H4M regulators (Additional file 1: Fig. S1D). Additionally, methylation levels of the majority of H4M regulators were lower in HCC samples compared to normal samples, which may contribute to the higher expression of these regulators in HCC tissues (Additional file 1: Fig. S1E).

Then, we elucidated widespread protein interactions that existed within the same type of regulator and between distinct types of regulators. Notably, the GO enrichment analysis revealed that these regulators are not only marked by histone methylation-related processes but also involved in immune response, cell cycle, and DNA damage repair. This may indicate the mechanisms of how these regulators affect the development of HCC (Fig. 1D). Based on the expression of 36 H4M regulators, HCC samples may be significantly distributed as either normal samples or HCC samples (Fig. 1E). This supports the notion that the expression pattern of H4M regulators may differ significantly between normal and HCC samples. Notably, except for PRMT6, the mRNA expression of other H4M regulators was significantly higher in HCC samples than in peritumoral tissues (Fig. 1F). The results of immunohistochemical staining confirmed the significant high expression of JMJD6, PHF8, PRMT3, PRMT5, RAD23A, SMARCA4, SMYD5, and TP53BP1 (Additional file 2: Fig. S2A-H). To some extent, the differential distribution of gene expression levels could be attributed to the variation of their copy number and DNA methylation changes [40]. In addition, the mRNA expression of the majority of H4M regulators differed significantly between four microenvironment subtypes and six immune-related subtypes (Additional file 1: Fig. S1F, Additional file 3: Fig. S3A; Additional file 14: Tables S5, S6) [41, 42]. Also, significant expression differences of 36 H4M regulators were found between HCC patients with TP53 mutation and those without TP53 mutation (Additional file 3: Fig. S3B, Additional file 14: Table S7). Then, numerous positive correlations were discovered between H4M regulators and the proliferation score, HRD score, and intratumor heterogeneity score, particularly between H4M regulators and the proliferation score (Additional file 3: Fig. S3C–E; Additional file 14: Table S8). These findings confirmed that these regulators play a crucial role in the microenvironment remodeling and malignant progression of HCC.

### H4M modification patterns mediated by 36 regulators

UniCox regression analysis showed that 24 H4M regulators were significantly related to the prognosis of HCC patients (Additional file 4: Fig. S4A). Integrative Kaplan–Meier analysis suggested that 30 H4M regulators, including ORC1 (Additional file 4: Fig. S4B) and FANCD2 (Additional file 4: Fig. S4C), were significantly associated with the overall survival of HCC patients, whereas only KDM4B was protective (Additional file 14: Table S9). The findings suggested that H4M modification was closely associated with HCC prognosis.

**Fig. 2:**
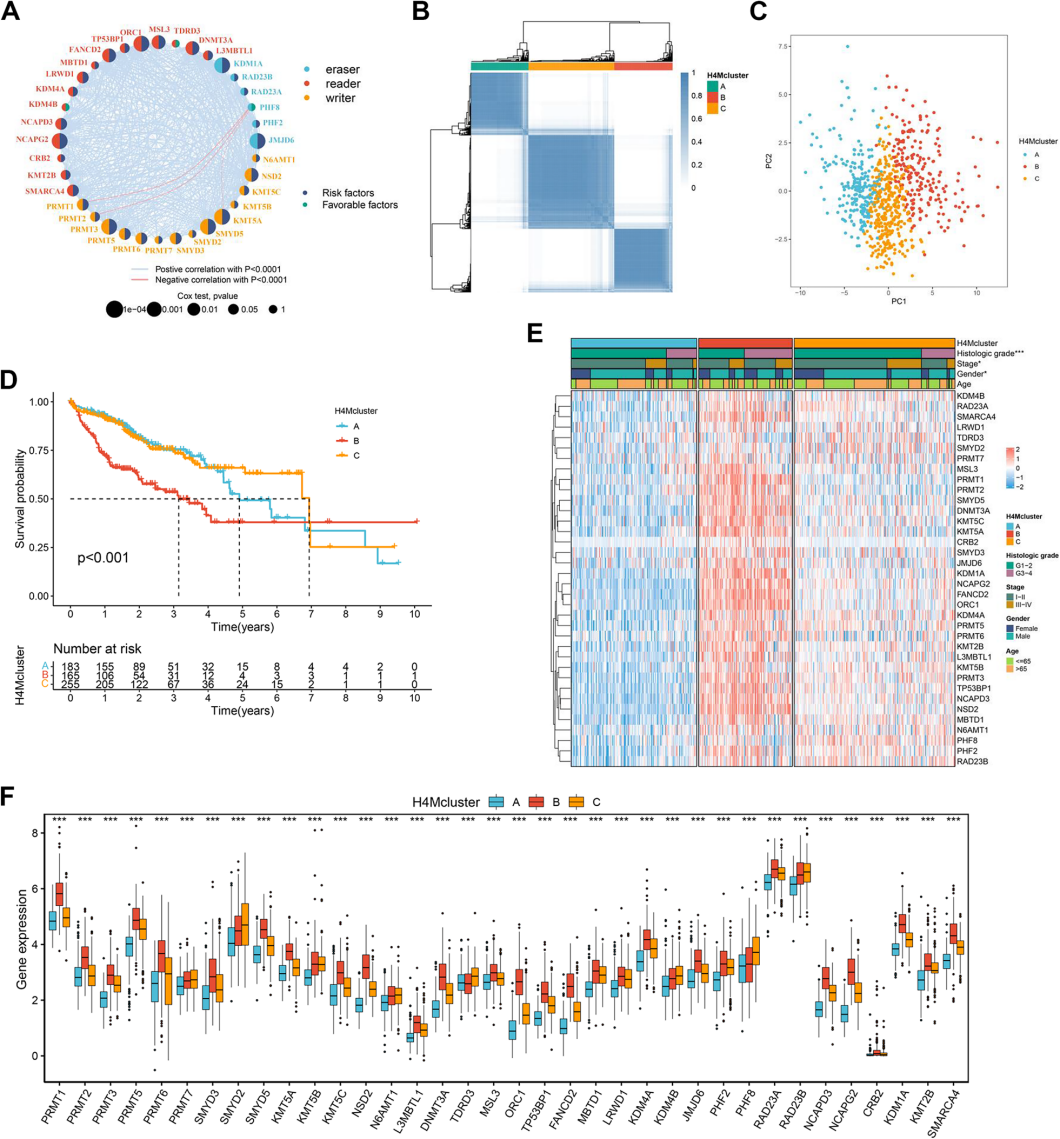
Three H4M modification patterns and relative biological functions. **A** A network of correlation including H4M regulators in the meta-data cohorts. **B** Consensus matrix heatmap defining three H4Mclusters (*k* = 3) and their correlation areas. **C** PCA analysis indicated a significant difference in transcriptomes between the three subgroups. **D** Kaplan–Meier survival analysis for H4Mclusters. **E** Differences in clinical characteristics and expression levels of H4M regulators between the three distinct H4Mclusters. **F** Detailed expression landscape of 36 H4M regulators between three H4M modification patterns. *, **, and *** mean *p* < 0.05, < 0.01, and < 0.001, respectively

Then, three RNA sequence data sets (TCGA-LIHC, ICGC-LICA, and ICGC-LIRI) were integrated into a meta-cohort for further analysis. Interestingly, the majority of the H4M regulators consistently exhibited positive correlations, with the exception of the correlation between PHF8 and PRMT1, PRMT2, and KMT5A (Fig. 2A). The largest correlation coefficient (0.82) was found between FANCD2 and ORC1 (Additional file 14: Table S10). Additionally, almost all other H4M regulators exhibited significantly higher expression in the ORC1 high expression group compared to the low expression group in the TCGA data set, except for TDRD3 (Additional file 4: Fig. S4D). This phenomenon could also be found between FANCD2 high and low expression groups (Additional file 4: Fig. S4E). These analyses demonstrated that the H4M regulators FANCD2 and ORC1 may serve as the hub of the entire network of H4M regulators in HCC. We further identified three potential modification patterns associated with H4M by conducting a consensus cluster algorithm, including 183 samples in pattern A, 165 samples in pattern B, and 255 samples in pattern C (Fig. 2B). These patterns were designated as H4Mcluster A-C, respectively (Additional file 14: Table S11). PCA analysis revealed that these patterns differed significantly (Fig. 2C). Kaplan–Meier analysis revealed survival probabilities varied significantly from the H4Mcluster to the H4Mcluster, with H4Mcluster-B having the lowest survival rate (Fig. 2D). To further verify the existence of three H4M modification patterns, 1034 HCC samples from eight external cohorts were integrated as a validation cohort (Additional file 5: Fig. S5A-B). It was found that these three H4M modification patterns could be fully validated (Additional file 5: Fig. S5C-D, Additional file 14: Table S12). The above results confirmed the presence of three potential H4M modification patterns.

### H4M modification pattern differences in clinical and biological features

Significant differences in clinical features were also found among the three patterns, including gender, TNM stage, and histologic grade (Fig. 2E). H4Mcluster-B exhibited the highest expression of 36 H4M regulators, which was consistent with its poor prognosis (Fig. 2F). The difference in constitutive and complementary hallmarks among the three patterns was revealed by GSVA analyses (Additional file 14: Table S13). Hallmarks enriched in H4Mcluster-A were relatively abundant in immune-related processes. While H4Mcluster-B was primarily associated with cancer pathways and genomic variation. For H4Mcluster-C, enriched hallmarks were mainly related to metabolism (Additional file 4: Fig. S4F, Additional file 6: Fig. S6A-B). The highest scores for intratumor heterogeneity, HRD, and proliferation were found in H4Mcluster-B (Additional file 6: Fig. S6C-E). It was hypothesized that H4Mcluster-A might have the strongest anti-tumor immune response, H4Mcluster-B may have more malignant biological behaviors, and H4Mcluster-C is intermediate. Further analyses revealed the differences in typical cancer signatures among the three H4M modification patterns (Additional file 14: Table S14). Immune responses were found to be significantly augmented in H4Mcluster-A, whereas signaling associated with cancer progression and immunosuppression was found to be predominantly enriched in H4Mcluster-B (Additional file 6: Fig. S6F-G), confirming our hypothesis.

### Tumor microenvironment and immune infiltration characteristics of distinct H4M modification patterns

**Fig. 3:**
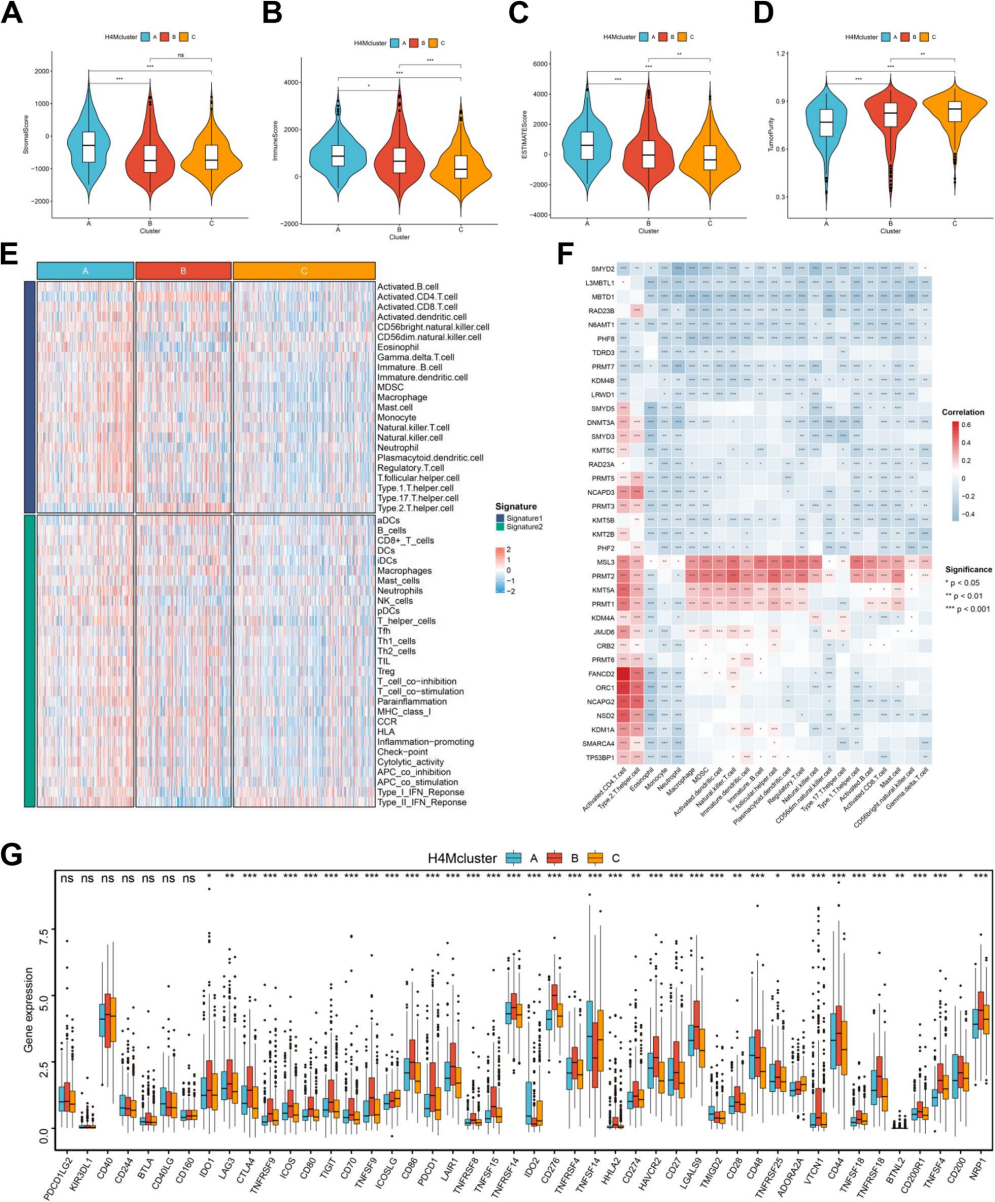
Correlations of tumor immune microenvironments and three H4M modification patterns in HCC. **A-C** Correlations between the three H4M modification patterns and TME score. **D** Correlations between the three H4M modification patterns and tumor purity. **E** Immune infiltration landscape in the three H4M modification patterns quantified by two types of immune-related signatures. **F** Correlation between the expression of 36 H4M regulators and the infiltration levels of 23 immune cells in TME. **G** Immune checkpoint differences among the three H4M modification patterns. *, **, and *** mean *p* < 0.05, < 0.01, and < 0.001, respectively

The TME plays a vital role in the occurrence and development of HCC. Based on the results of the ESTIMATE algorithm, we found a tremendous difference in HCC TME among three H4M modification patterns, whether in the aspect of stromal or immune components (Fig. 3A–C). Moreover, the three H4M modification patterns exhibited a substantial disparity in tumor purity (Fig. 3D). The above results indicated the pivotal role of H4M modification in TME remodeling. Further analyses revealed robust differences in immune infiltration in HCC TME (Fig. 3E, Additional file 14: Table S15). According to the constitution of immune cells, three H4M modification patterns presented definite immune infiltration characteristics. H4Mcluster-A had an immune-inflamed phenotype, with adaptive immune cell infiltration and immune activation; H4Mcluster-B had an immune-suppressed phenotype, with abundant CD4^+^ T cell and Th2 cell infiltration; and H4Mcluster-C was somewhere in the middle (Additional file 7: Fig. S7A). Then, another group of reported immune cells and immune function markers demonstrated similar enrichment differences between three H4M modification patterns (Additional file 7: Fig. S7B), reinforcing the finding. Correlation analysis between 36 regulators and infiltrating immune cells further confirmed that histone H4M modification was closely associated with immune infiltration in TME (Fig. 3F). Except for MSL3, PRMT2, KMT5A and PRMT1, most H4M-related regulators were negatively correlated with immune infiltration in TME, including CD8^+^ T cells and Th1 cells. These genes with a positive regulatory function may contribute to the promotion of anti-tumor immune response. Also, previous reports have confirmed that the TME always be immunosuppressive, and Th1/Th2 balance in CD4^+^ T cells is always dominated by the Th2 phenotype [43, 44]. In the present study, activated CD4^+^ T cells and Th2 cells were found to be significantly positively related to most H4M regulators. These results indicated that H4M modification-mediated TME characteristics might generally be suppressed. Furthermore, we analyzed the gene expression differences in immune checkpoints among the three H4M modification patterns. Most immune checkpoint genes were found to be significantly overexpressed in H4Mcluster-B (Fig. 3G). This result indicated that H4Mcluster-B had greater immune tolerance and suppression, which was consistent with earlier findings.

### Construction of co-expression networks and identification of H4M modification patterns-associated gene co-expression modules

**Fig. 4:**
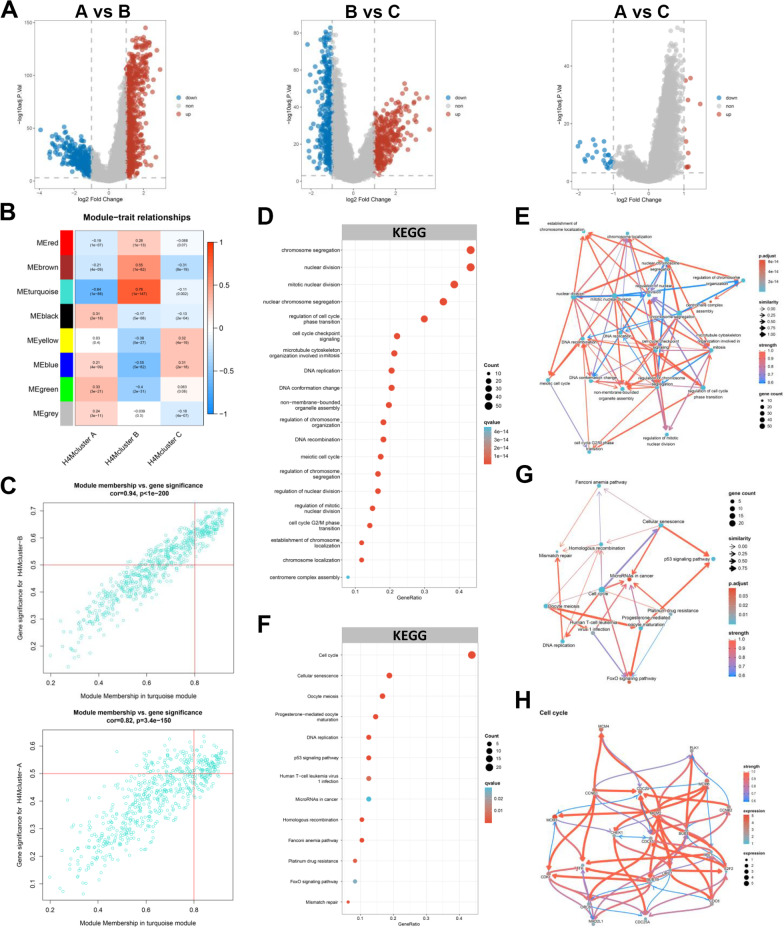
Identification of hub genes and functional annotation. **A** Differential analysis among three H4M modification patterns. **B** Heat map of the correlations between the module eigengenes and H4M modification patterns. The Pearson correlation coefficient and corresponding p value were displayed in each cell. **C** Scatterplots of correlation between MEturquoise membership and gene significance for H4Mcluster-B and H4Mcluster-A. **D** KEGG enrichment analysis for 133 hub genes identified by WGCNA analysis. **E** Enriched pathway regulatory network for 20 pathways. **F** KEGG enrichment analysis for 39 feature genes identified by LASSO regression analysis. **G** Enriched pathway regulatory network for 13 pathways. **H** The gene regulatory network for the cell cycle signaling pathway

According to the results of the difference analysis, 1084 DEGs were identified between H4Mcluster-A and H4Mcluster-B, 665 DEGs were identified between H4Mcluster-B and H4Mcluster-C, and 34 DEGs were identified between H4Mcluster-A and H4Mcluster-C (Fig. 4A). After the removal of the overlapping genes, a total of 1223 genes were defined as DEGs characterizing H4M modification patterns. A total of 7 distinct gene co-expression modules were generated by WGCNA analysis. The detailed analysis process is presented in (Additional file 7: Fig. S7C-E). The results confirmed that the turquoise module exhibited the strongest positive correlation with the H4Mcluster-B phenotype (*R* = 0.76, *p* = 1e-147) and negative correlation with the H4Mcluster-A (*R* = − 0.64, *p* = 1e-88) (Fig. 4B). Then, 133 shared hub genes were extracted based on the gene significance and module membership correlation scatterplots (Fig. 4C, Additional file 7: Fig. S7F). Pathway analysis revealed that they were predominantly enriched in cell cycle-related processes, which highlights the effects of H4M modification on tumor proliferation (Fig. 4D). Cell cycle checkpoint signaling played a pivotal role in the entire predicted regulatory network (Fig. 4E). Notably, the results of the uniCox regression analysis demonstrated that all 133 genes were significantly associated with the adverse prognosis of HCC patients (Additional file 14: Table S16).

### Validation of H4M modification patterns and construction of the H4M scoring system

**Fig. 5:**
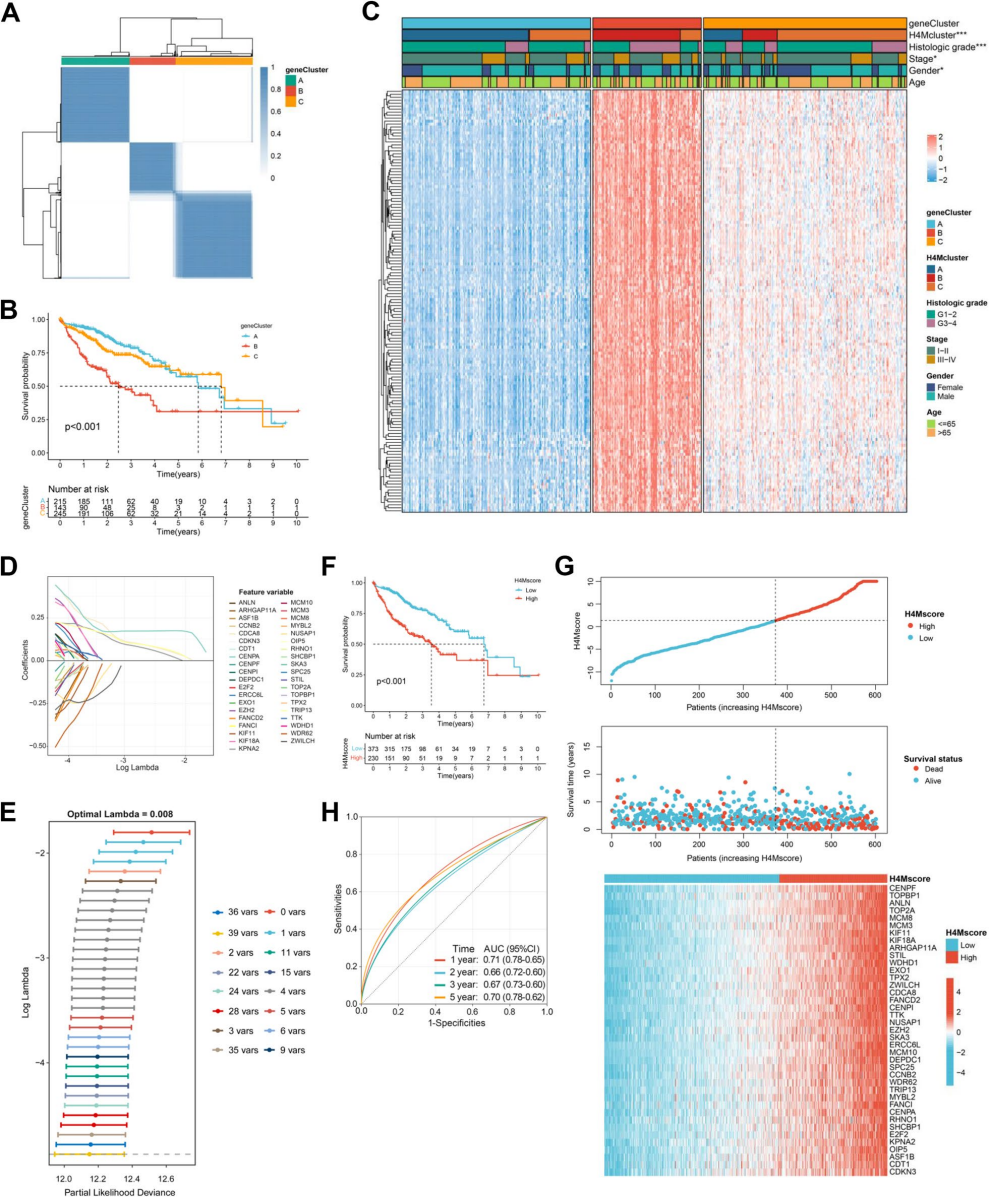
Construction of H4M signature. **A** Consensus matrix heatmap defining three geneClusters (*k* = 3) and their correlation areas. **B** Kaplan-Meier survival analysis for H4M geneClusters. **C** The H4M modification patterns and other clinical characteristics were used as patient annotations. Differences in the expression levels of 133 hub genes were exhibited between the three distinct geneClusters. **D-E** The process of filtering genes by LASSO regression model. **F** Kaplan–Meier survival analyses for the high-H4Mscore and low-H4Mscore groups. **G** H4Mscore distribution, survival status of patients, and gene expression of 39 feature genes in H4M-related signature. **H** ROC curves measuring the predictive value of the H4Mscore signature. *, **, and *** mean *p* < 0.05, < 0.01, and < 0.001, respectively

To further comprehend the distinct effects generated by H4M modification in HCC, based on 133 selected DEGs, we successfully categorized HCC samples into three distinct genomic phenotypes via unsupervised clustering analysis and termed them H4M geneCluster A-C, respectively (Fig. 5A). Survival analysis suggested the relatively worst prognosis in the H4M geneCluster-B (Fig. 5B). As expected, multiple clinical parameters exhibited differential distribution among three distinct genomic phenotypes and most of the 36 H4M regulators were also overexpressed in the H4M geneCluster-B (Fig. 5C, Additional file 7: Fig. S7G). In addition, the intratumor heterogeneity score, the HRD score, and the proliferation score were significantly higher in the H4M geneCluster-B, confirming its greater propensity for malignancy (Additional file 7: Fig. S7H-J). Collectively, robust differences were discovered between three distinct genomic phenotypes, whether in the landscape of clinical traits or degree of malignancy, which profoundly confirmed the three H4M modification patterns described previously.

Considering the heterogeneity of HCC and the diversity of H4M modification, it was decided to construct a scoring system to quantify the H4M modification levels of individual cases. At first, the LASSO regression model was applied to 133 filtered hub DEGs (Fig. 5D). Consequently, 39 feature genes were screened out (Fig. 5E). In the regulation network comprised of 39 feature genes, the cell cycle pathway is centrally located. Internal gene regulatory network in the cell cycle pathway was also inferred (Fig. 4G-H). Then, a 39-gene H4Mscore system was constructed using the PCA algorithm. In accordance with the prominent prognosis of H4Mcluster-B and H4M geneCluster-B, these two groups owned the highest H4Mscore (Additional file 8: Fig. S8A-B). These results demonstrated that H4Mscore could effectively reflect the H4M modification characteristics of individual HCC patients.

### Prognosis and biological function relevance of H4Mscore in HCC

**Fig. 6:**
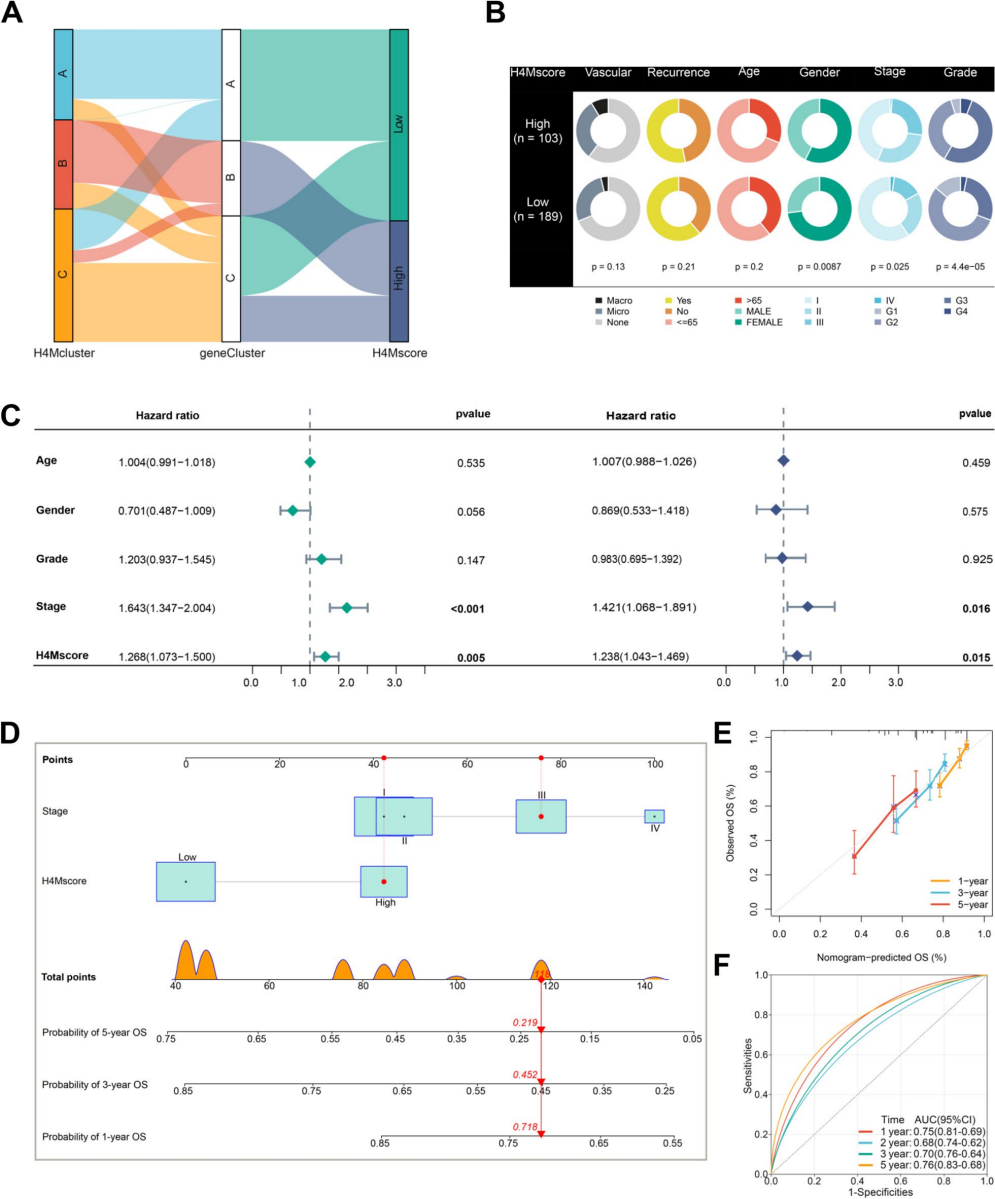
Clinical features of the distinct H4Mscore groups and nomogram construction. **A** Sankey plot showing the changes of H4Mclusters, H4M geneClusters, and H4M score. **B** Clinical characterization in low and high H4Mscore groups. The chi-square test was used to calculate statistical differences. **C** The uniCox and multiCox regression analyses of H4Mscore and clinical features. **D** The nomogram for predicting 1-, 3-, and 5-year OS based on H4Mscore signature and clinical stage. OS, overall survival. **E** Calibration for detecting nomogram at 1-, 3-, and 5-year OS. **F** The 1-, 2-, 3-, and 5-year time-dependent ROC curves for evaluating the nomogram

According to the Kaplan-Meier survival analysis, all HCC patients were divided into high- and low-H4Mscore groups, with the high-H4Mscore group having a significantly poorer survival outcome (Fig. 5F). As the H4M score rises, the number of HCC-related deaths gradually increases. The 39 feature genes have relatively higher expression in the high-H4Mscore group (Fig. 5G). The area under curves (AUC) of ROC analysis for 1, 2, 3 and 5 year were 0.71, 0.66, 0.67 and 0.70, respectively (Fig. 5H). Also, this prediction for survival outcome could be validated in different subgroups (Additional file 8: Fig. S8C-L). Surprisingly, eight external HCC cohorts confirmed the survival difference between groups with a high H4Mscore and those with a low H4Mscore (Additional file 9: Fig. S9A-H). To further validate the accuracy of the scoring system, we constructed a network of protein–protein interactions between 39 feature genes (Additional file 10: Fig. S10A). Then, seven core genes were selected by 12 algorithms for experimental validation (Additional file 10: Fig. S10B). It was found that the expression of these seven core genes was higher than that in adjacent tissues in the TCGA database (Additional file 10: Fig. S10C). Kaplan–Meier analysis showed that these genes were extremely related to the poor survival outcome of HCC patients (Additional file 10: Fig. S10D-J). The results of qPCR suggested that all these genes were overexpressed in HCC tissues than adjacent tissues (Additional file 11: Fig. S11A-G). Consistently, relative siRNAs markedly inhibited the expression of seven core genes and decreased the proliferation and migration ability of HCC cell line (Additional file 12: Fig. S12A-I). The aforementioned results confirmed that H4Mscore was an excellent prognostic marker and had an extremely stable prediction efficiency for prognosis in HCC. As shown in the Sankey diagram, most patients in H4Mcluster-B belonged to H4M geneCluster-B and high-H4Mscore group, and patients from H4M geneCluster-B occupied a larger proportion in the high-H4Mscore group than in the low-H4Mscore group (Fig. 6A). In terms of clinical characteristics, the low-H4Mscore group had a higher proportion of HCC patients in advanced or poorly differentiated states (Fig. 6B). Further uniCox analysis revealed that H4Mscore and TNM stage were adverse prognostic factors, and multiCox analysis ulteriorly confirmed that H4Mscore and TNM stage were independent prognostic indicators (Fig. 6C). Combining multiple clinical data, we developed a nomogram to accurately predict the 1-, 3-, and 5-year survival rates of HCC patients (Fig. 6D). The calibration diagram confirmed the nomogram's predicted value was close to reality (Fig. 6E). ROC analysis for the nomogram suggested the nomogram had a prominent efficiency in predicting prognosis of 1, 2, 3, and 5 years and the AUCs were 0.75, 0.68, 0.70, and 0.76, respectively (Fig. 6F).

**Fig. 7:**
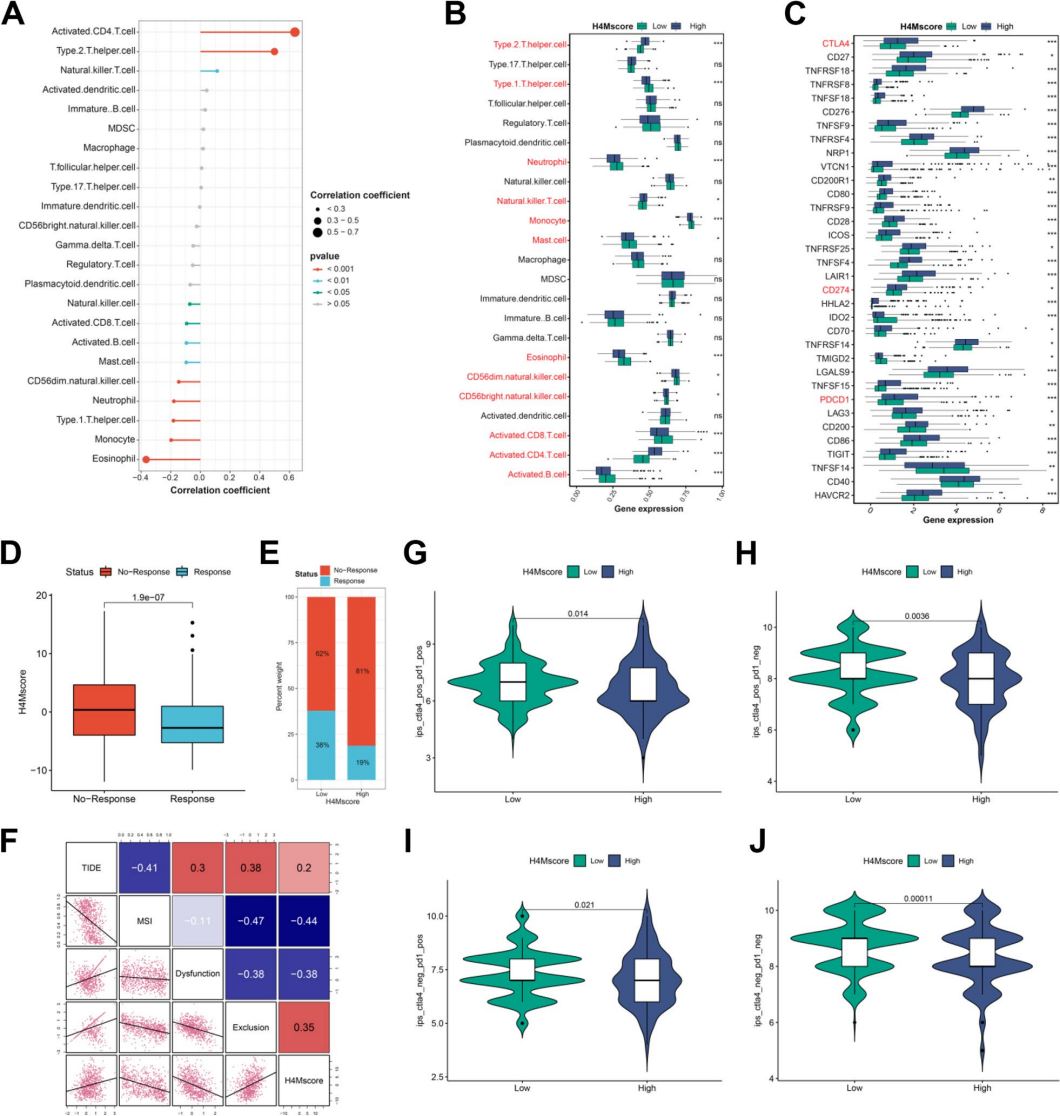
The role of H4M modification patterns in immune infiltration and the response to immunotherapy. **A** Correlation between H4Mscore and immune infiltration. **B** Differences in immune infiltration between high and low H4Mscore groups. **C** Differences in immune checkpoints between high and low H4Mscore groups. **D** Differences in H4Mscore between the response and no response groups based on the TIDE database. **E** The proportion of patients who responded to immune checkpoint blocker therapy in the low or high H4Mscore groups. **F** Correlation analysis of H4Mscore and multiple immunotherapy markers. **G-J** The immunophenoscore differences between high and low H4Mscore groups. *, **, and *** mean p < 0.05, < 0.01, and < 0.001, respectively

Additional analysis revealed that H4Mscore correlates positively with three cancer signatures, particularly the proliferation score (*R* = 0.91) (Additional file 13: Fig. S13A-C). Moreover, nearly all signatures associated with cancer progression demonstrated a robust distinction, and the majority of these signatures were enriched in the high-H4Mscore group (Additional file 13: Fig. S13D). Then, we detected differences in additional signature groups associated with cancer pathways and processes. It was found that most cancer-related pathways and phenotypes were primarily enhanced in the high-H4Mscore group (Additional file 13: Fig. S13E). This result was generally in accordance with the above signature and additionally suggested vast differences in cancer pathways. The stemness indices based on transcriptome data could effectively measure the level of tumor stemness [39]. In the present study, it was determined to be a hazardous factor for the survival of patients with HCC and to have a significant positive correlation with H4Mscore (*R* = 0.43) (Additional file 13: Fig. S13F, Additional file 14: Table S17). The results confirmed that the cancer stemness of the group with a high H4Mscore was stronger. Evidently, combined survival analysis revealed that enhanced cancer stemness negatively affected the prognosis of HCC patients, but it had a lower risk degree than a high H4Mscore (Additional file 13: Fig. S13G). We further analyzed the somatic mutation differences based on the differences in genomic signatures and found a higher mutation frequency in high-H4Mscore groups (Additional file 13: Fig. S13H). Moreover, the low-H4Mscore group had relatively stronger cytolytic activity and weaker immune suppression than the high-H4Mscore group, which indicated that the anti-tumor immune response was significantly fiercer in the low-H4Mscore group (Additional file 13: Fig. S13E). Significantly positive correlations were observed between H4Mscore and activated CD4^+^ T cells, as well as between H4Mscore and Th2 cells (Fig. 7A). Similarly, significant differences in immune infiltration were discovered between groups with high and low H4Mscores. Immune suppression-related cells (activated CD4^+^ T cells and Th2 cells) were extremely enriched in the high-H4Mscore group, which was consistent with its stronger immune suppression score (Fig. 7B). Also, immune checkpoints presented significant differences between high-H4Mscore and low-H4Mscore groups, including PDCD1 (PD-1), CD274 (PD-L1), and CTLA4 (Fig. 7C). The aforementioned study confirmed our hypothesis that individuals with a high H4Mscore had a more potent immune suppressive effect.

### The role of the H4Mscore in anti-PD-1/L1 immunotherapy

It has been demonstrated that blocking immune checkpoints such as PD-1, CTLA4 and PD-L1 is promising and has led to significant advances in the treatment of HCC. In this study, significant differences in immune response-related signatures and immune infiltration have been confirmed between groups with high and low H4Mscores. It was further speculated that these two groups of HCC patients would respond differently to immunotherapy. The group with a low H4Mscore had a greater proportion of patients who responded to anti-PD-1 and anti-CTLA4 immunotherapy, and the H4Mscore was higher in nonresponding HCC patients (Fig. 7D–E). Further analysis revealed that TIDE and immune exclusion score had significant positive correlations with H4Mscore (Additional file 14: Table S18). Meanwhile, significant negative correlations were found between H4Mscore and MSI score, which indicated the genome of the low-H4Mscore group was relatively unstable and was more likely to produce tumor neoantigen (Fig. 7F). These results based on the TIDE database suggested that anti-PD-1 and anti-CTLA4 immunotherapy were more effective in the low-H4Mscore group. Regardless of anti-PD-1 and anti-CTLA4 monotherapy or combination therapy, the group with a lower H4Mscore demonstrated superior efficacy (Fig. 7G-J; Additional file 14: Table S19). These results preliminarily confirmed our conjecture.

**Fig. 8:**
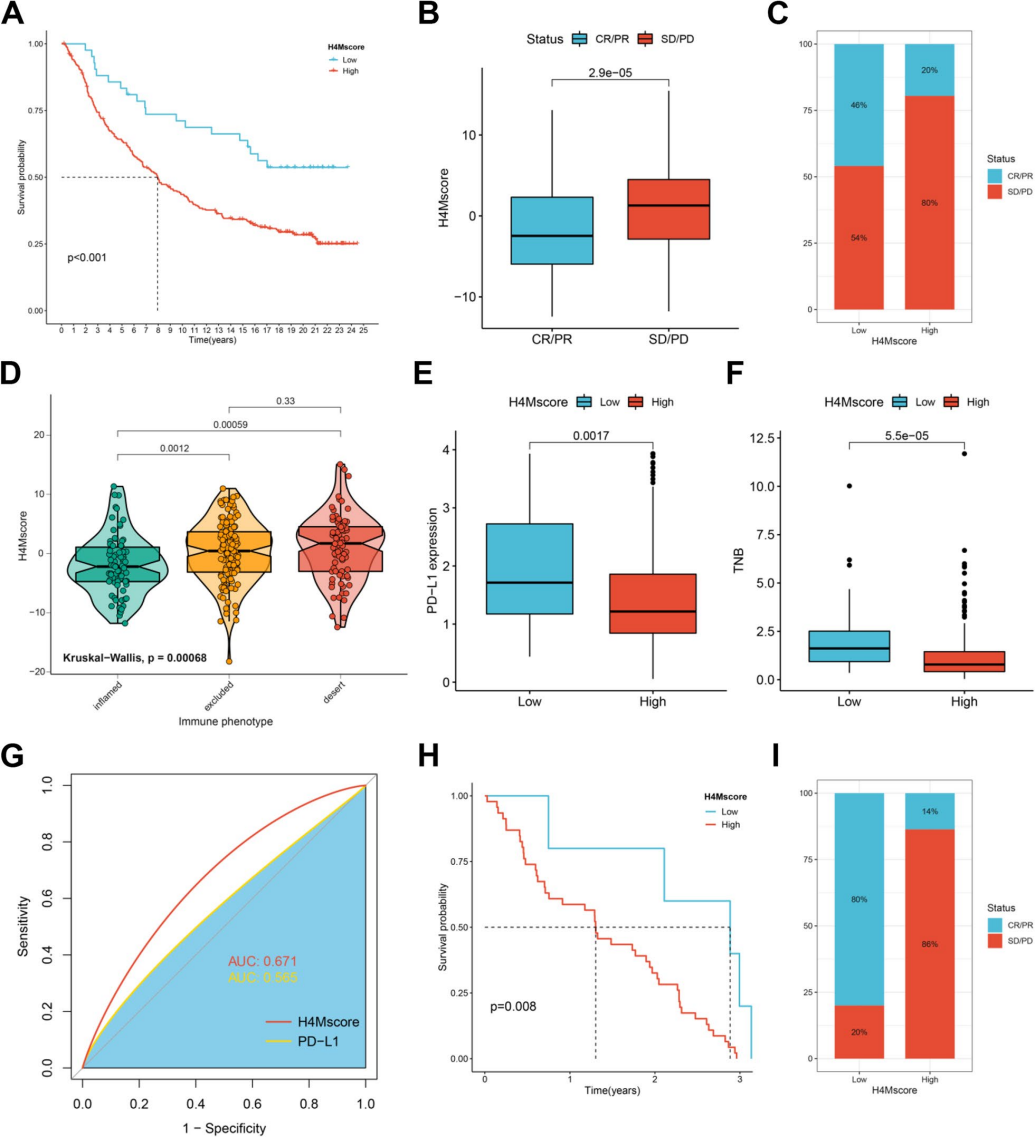
Verifying the potential of H4Mscore as an immunotherapy biomarker in external immunotherapy cohorts. **A** Kaplan–Meier survival analyses for the high H4Mscore and low H4Mscore groups in the IMvigor210 cohort. **B** The H4Mscore difference between the CR/PR and SD/PD groups. CR, complete response; PR, partial response; SD, stable disease; PD, progressive disease. **C** The proportion of patients who responded to immunotherapy in the low or high H4Mscore groups. **D** Differences in H4Mscore among distinct tumor immune phenotypes in the IMvigor210 cohort. **E** Differences in PD-L1 expression between high and low H4Mscore groups. **F** Differences in tumor neoantigen burden (TNB) between high and low H4Mscore groups. **G** Predictive value of the H4Mscore and PD-L1 expression for immunotherapy response. **H** Kaplan–Meier survival analyses for the high and low H4Mscore groups in the GSE91061 cohort. **I** The proportion of patients that responded to immunotherapy in the low or high H4Mscore groups in the GSE91061 cohort

Then, we further investigated the value of H4Mscore in predicting response to multiple immunotherapies. Regretfully, there was a lack of an open-access HCC immunotherapy cohort with complete survival parameters and transcriptome data. For the time being, we could only confirm the utility of the H4Mscore in other cancer cohorts. In the IMvigor210 cohort, an anti-PD-L1 metastatic urothelial cancer data set, survival analysis suggested patients with a higher H4Mscore exhibited the worst prognosis and a lower response rate to immunotherapy (Fig. 8A–B). Next, we analyzed the relationship between H4Mscore and different response statuses. It was found that patients with a disease release had a lower H4Mscore than those with disease stability or progression (Fig. 8C). According to the study by Daniel S., tumors could be distinguished into three immunophenotypes with significant immune infiltration and immunotherapy response differences, including immune-inflamed phenotype, immune–excluded phenotype, and immune-desert phenotype [45]. H4Mscore was found to be lowest in the immune-inflamed phenotype and highest in the immune-desert phenotype, which was consistent with their immunotherapy response (Fig. 8D). The level of PD-L1 expression had a certain value in predicting immunotherapy response, and it was found to be higher expressed in the low-H4Mscore group (Fig. 8E). Tumor neoantigen is produced by the genetic mutation of tumor cells and is only expressed in tumor cells, which has been demonstrated to be a crucial marker for predicting the immunotherapy response [46, 47]. In our study, tumor neoantigen burden indicated a markedly prolonged survival and was found to be extremely higher in the low-H4Mscore group (Fig. 8F). Furthermore, the ROC curve revealed that H4Mscore had a better predictive effect on immunotherapy response than PD-L1 (Fig. 8G). Patients with a low H4Mscore had a significant survival advantage in another melanoma cohort treated with anti-PD-1 and anti-CTLA4 inhibitors (GSE91061) (Fig. 8H). Similarly, patients with a low H4Mscore had an increased response rate, which was in accordance with the findings of the IMvigor210 cohort (Fig. 8I).

In conclusion, H4M modification was significantly correlated with HCC immunophenotypes, and H4Mscore was an excellent biomarker for predicting immunotherapy response.

## Discussion

Increasing evidence has enhanced our comprehension of the landscape of H4M modification in cancer occurrence and development, sparking our interest in the role of H4M modification in HCC. Nevertheless, the majority of the previous studies merely focused on single H4M regulators or single biological processes. The interactions between H4M regulators and their overall effect on the TME and anti-tumor immunity need to be further elucidated. Identifying the practical role of H4M modification in the TME will contribute to a better understanding of the mechanism of HCC progression and help guide more effective immunotherapy strategies.

Based on 36 H4M regulators, three distinct H4M modification patterns were identified. H4Mcluster-A had numerous immune cell infiltrations in TME, which was characterized by an immune-inflamed phenotype. Patients in H4Mcluster-A may have a better immune checkpoint blocker response [45, 48]. H4Mcluster-B was characterized by a phenotype of immune suppression. Although patients split into H4Mcluster-B also had abundant immune cell infiltration, they could not induce normal anti-tumor immunity [6, 37, 45]. H4Mcluster-C was intermediate, and this modification pattern didn't reveal specific immune infiltration characteristics. In addition, we constructed an H4M-related scoring system, which could effectively reflect H4M modification in an individual tumor sample.

Immune checkpoint blockade can elicit robust and long-lasting responses, showing promise as one of the principal therapeutic modalities in patients with various cancers [49, 50], including HCC [51]. However, no biomarker has been validated accurately to guide clinical decision-making [52]. The H4M scoring system was found to be significantly correlated with immune cell infiltration and immune checkpoint expression, indicating a strong potential to predict immunotherapy response. Multiple reported indicators confirmed the pivotal role of H4Mscore in predicting the response to immunotherapy, including the immunophenoscore and TIDE score. The high H4Mscore was identified as a risk factor for immunotherapy in patients with HCC and was associated with a poorer immunotherapy response. In addition, external immunotherapy cohorts were used to validate this finding. In IMvigor210 and GSE91061 cohorts, patients with a higher H4Mscore suggested a worse prognosis and lower sensitivity to immunotherapy. Amazingly, H4Mscore was superior to PD-L1 in distinguishing patients who responded to immunotherapy. This demonstrates conclusively that the H4Mscore-related system has significant clinical utility for predicting immunotherapy response.

The TME, which is made up of tumor cells and stromal cells, exhibits an immunosuppressive trait in HCC patients, which not only induces tumor progression but also poses a big challenge for an effective anti-tumor immune response [53]. Also, it has been demonstrated that the clinical response to immunotherapy is strongly correlated with the tumor immune microenvironment of HCC [52]. Three different H4M modification patterns could distinguish TME into different statuses. According to the expression landscape of H4M regulators, we could speculate on paired TME features, which is helpful in understanding the mechanism of HCC progression and predicting the immunotherapy response.

Many published works have highlighted the vital role of H4M in various cancers, and for instance, PHF8 played an oncogene function and contributed to EMT and metastasis in HCC [54, 55]. KDM1A was found to facilitate immunosuppression by upregulating PD-L1 expression and KDM1A-targeting therapy could reduce acquired resistance to sorafenib and improve HCC therapeutic efficacy [56, 57]. Different from previous studies, we elucidated the landscape of H4M regulators via multi-omics analysis in HCC for the first time. Whether at the genome or transcriptome level, H4M regulators exhibited robust differences between HCC and normal samples.

Briefly, the cluster results of the H4M patterns and H4M geneClusters are comparable, indicating that our cluster method is reliable, elucidates the heterogeneity of tumors in-depth, and augments the existing classification systems. The H4Mscore can comprehensively evaluate the H4M modification pattern of specific patients and corresponding TME characteristics, further identifying tumor immunological traits and guiding more effective clinical decision-making. The H4Mscore can also be utilized as a reliable prognostic biomarker to predict survival and the effectiveness of immunotherapy. Eight external HCC cohorts and two classical immunotherapy cohorts confirmed our conclusion. Nevertheless, due to the lack of an HCC cohort treated with immunotherapies, more prospective trials are expected to validate these findings further. In summary, our findings provided novel ideas for improving the clinical responses of patients to immunotherapy, identifying different TME features, and promoting personalized cancer immunotherapy in future.

## Conclusion

In conclusion, H4M modifications contributed to the complexity and diversity of TME. The H4Mscore could reflect the TME and immunological status of individual patients, which could accurately predict prognosis and facilitate clinical transformation in HCC.

## Supplementary Information

**Additional file 1**. Figure S1: Correlation analyses for H4M regulators expression and CNV and methylation levels. **A** The mutation co-occurrence and exclusion analysis for histone methylation modification (H4M) regulators in the TCGA-LIHC cohort. Co-occurrence: aquamarine; exclusion: claybank. **B** The location of H4M modification genes on 23 chromosomes in TCGA-LIHC. **C** Correlation between mRNA expression and CNV variation levels of H4M regulators. **D** Correlation between mRNA expression and methylation levels of H4M regulators. **E** The differences in methylation level of H4M regulators between tumor and normal samples. **F** Expression of 36 H4M regulators between four microenvironment subtypes. IE/F: immune-enriched and fibrotic; IE: immune-enriched but non-fibrotic; F: fibrotic; D: immune-depleted. *, **, and *** mean p < 0.05, < 0.01, and < 0.001, respectively.

**Additional file 2**. Figure S2：Immunohistochemistry of H4M regulators. **A-H** The protein levels of H4M regulators in normal liver and LIHC were visualized by immunohistochemistry in HPA.

**Additional file 3**. Figure S3: The expression landscape of H4M regulators in different subtypes and its correlation with different cancer markers. **A** Expression of 36 H4M regulators between six immune subtypes. **B** Expression of 36 H4M regulators between p53 wild and mutation groups. Three cancer signatures were involved, including homologous recombination deficiency (HRD), intratumor heterogeneity, and proliferation score. The correlation between these three cancer signatures and H4M writers was determined. **C**, H4M erasers **D**, and H4M readers **E**, respectively. *, **, and *** mean p < 0.05, < 0.01, and < 0.001, respectively.

**Additional file 4**. Figure S4: Prognostic analysis for H4M regulators and the importance of ORC1 and FANCD2 in overall H4M modification. **A** Expression of H4M regulators between ORC1 high expression group and low expression group. **B** Expression of H4M regulators between FANCD2 high expression group and low expression group. **C** The forest plot of the HR for the correlation between H4M regulators and the prognosis of HCC patients. **D** Kaplan–Meier survival analyses for the high and low ORC1 expression groups. **E** Kaplan–Meier survival analyses for the high and low FANCD2 expression groups. **F** The difference in enriched hallmarks between H4Mcluster-A and H4Mcluster-C. *, **, and *** mean p < 0.05, < 0.01, and < 0.001, respectively.

**Additional file 5**. Figure S5: Three H4M modification patterns were validated in the integrated external cohort. **A** The distribution of eight data sets before consolidation. **B** The distribution of eight data sets after removing the batch effects. **C** Consensus matrix heatmap defining three H4Mclusters (k = 3) and their correlation areas. **D** Kaplan–Meier survival analysis for H4Mclusters.

**Additional file 6**. Figure S6: Biological function differences among three distinct H4M modification patterns. **A** Difference in enriched hallmarks between H4Mcluster-A and H4Mcluster-B. **B** Difference in enriched hallmarks between H4Mcluster-B and H4Mcluster-C. **C** Differences in intratumor heterogeneity score among three H4Mclusters. **D** Differences in HRD score among three H4Mclusters. **E** Differences in proliferation score among three H4Mclusters. **F-G** Two groups of typical cancer signatures differences among three H4Mclusters. *, **, and *** mean p < 0.05, < 0.01, and < 0.001, respectively.

**Additional file 7**. Figure S7: Immune infiltration evaluation and WGCNA analysis process. **A-B** Evaluating tumor-infiltrating immune cell abundance differences among three H4Mclusters. **C-E** The detailed analysis process of identifying key modules by WGCNA analysis. **F** Scatterplots of correlation between MEturquoise membership and gene significance for H4Mcluster-C. **G** Expression of 36 H4M regulators among three H4M geneClusters. **H-J** Differences in intratumor heterogeneity score, HRD score, and proliferation score among three H4M geneClusters. *, **, and *** mean p < 0.05, < 0.01, and < 0.001, respectively.

**Additional file 8**. Figure S8: Validating the prognostic value of H4Mscore in the internal and external cohorts. **A-C** Kaplan–Meier survival analyses for the high and low H4Mscore groups in three internal cohorts, including TCGA, ICGC-LIRI, and ICGA-LICA, respectively. **D-H** Kaplan–Meier survival analyses for the high and low H4Mscore groups in five external cohorts, including NODE-OEZ005255, GPL3921-GSE14520, GSE76427, GSE116174, and GPL571-GSE14520, respectively.

**Additional file 9**. Figure S9: Validating the prognostic value of H4Mscore in different clinical subtypes. **A-B** Difference in H4Mscore among three H4Mclusters and three H4M geneClusters, respectively. **C-L** Kaplan–Meier survival analyses for the high and low H4Mscore groups in different subtypes, including age, gender, histologic grade, TNM stage, and vascular invasion, respectively.

**Additional file 10**. Figure S10: Selection of hub genes for further experiments and validation. **A** The protein–protein interaction network. **B** The hub genes are selected by 12 cytoHubba algorithms in the Cytoscape software. **C** Expression of seven core genes between normal and HCC samples. **D-J** The Kaplan–Meier survival analyses for seven filtered hub genes. *, **, and *** mean p < 0.05, < 0.01, and < 0.001, respectively.

**Additional file 11**. Figure S11: Expression of seven core genes in clinical hepatocellular carcinoma (HCC) samples and paired paracancerous samples. **A-G** mRNA expression of seven core genes in HCC samples (T) and paracancerous samples (P). *, **, and *** mean p < 0.05, < 0.01, and < 0.001, respectively.

**Additional file 12**. Figure S12: Effects of hub genes on the proliferation and migration of HCC cells. After being treated with siRNAs, the mRNA expression and optical density curves of CCNB2 (**A**), CDCA8 (**B**), CENPF (**C**), EXO1 (**D**), TOP2A (**E**), TPX2 (**F**), and TTK (**G**) in Huh‐7 cells. **H-I** Migration experiment of Huh‐7 cells treated with siRNA for 48 hours. Cells were stained with crystal violet. Scale bar, 100 μM. *, **, and *** mean p < 0.05, < 0.01, and < 0.001, compared with negative control (NC) group.

**Additional file 13**. Figure S13: Correlation between H4Mscore and cancer signatures. By employing the Spearman method, correlation analyses between H4Mscore and HRD score (**A**), intratumor heterogeneity score (**B**), and proliferation score (**C**) were performed. (**D-E**) Two groups of typical cancer signatures differences between the high H4Mscore and low H4Mscore groups. (**F**) Correlation analyses between H4Mscore and mRNA stemness index (mRNAsi) using Spearman method. (**G**) Survival analyses for HCC patients stratified by both H4Mscore and mRNAsi using Kaplan–Meier curves. (**H**) The waterfall plot of tumor somatic mutation established by those with high H4Mscore and low H4Mscore. *, **, and *** mean p < 0.05, < 0.01, and < 0.001, respectively.

**Additional file 14**. Table S1. Basic information of hepatocellular carcinoma (HCC) datasets and external immunotherapy datasets included in this study. Table S2. Source and function of 36 histone H4 methylation (H4M) related regulators. Table S3. Primer sequences for qPCR. Table S4. The siRNA duplex sequences for functional experiments. Table S5. Microenvironment subtypes of HCC patients in TCGA. Table S6. Immune subtypes of HCC patients in TCGA. Table S7. TP53 mutation status of HCC patients in TCGA. Table S8. Intratumor heterogeneity score, proliferation score and Homologous Recombination Defects (HRD) score of HCC patients in TCGA-LIHC cohort. Table S9. UniCox regression and KM analyses for 36 H4M regulators. Table S10. Correlation analysis for 36 H4M regulators in meta-cohort. Table S11. Samples clustering in HCC RNA-seq meta cohorts. Table S12. Samples clustering in integrated external HCC cohorts. Table S13. Enrichment score of hallmark gene sets in HCC RNA-seq meta cohorts by GSVA analysis. Table S14. Enrichment score of HCC RNA-seq meta cohorts in cancer-related signatures. Table S15. Enrichment score of HCC RNA-seq meta cohorts in immune cell infiltration or immune signatures. Table S16. UniCox regression analysis for 133 hub genes. Table S17. The mRNA stemness index (mRNAsi) of HCC RNA-seq meta cohorts. Table S18. Immune response prediction by TIDE database. Table S19. Immunophenotype score predicted by TCIA database.

## Data Availability

TCGA, ICGC, GEO, and NODE belong to public databases. Two immunotherapy cohorts are also open. Users can download relevant data for free for research and publish relevant articles.

## Abbreviations

H4M: H4 methylation
TME: Tumor microenvironment
HCC: Hepatocellular carcinoma
TCGA: The Cancer Genome Atlas
ICGC: International Cancer Genome Consortium
CNV: Copy number variation
GEO: Gene Expression Omnibus
NODE: National Omics Data Encyclopedia
GSVA: Gene set variation analysis
MSigDB: Molecular signatures database
DEGs: Differentially expressed genes
WGCNA: Weighted gene co-expression network analysis
GO: Gene ontology
KEGG: Kyoto Encyclopedia of Genes and Genomes
LASSO: Least absolute shrinkage and selection operator
PCA: Principal component analysis
TIDE: Tumor immune dysfunction and exclusion
MSI: Microsatellite instability
