# Supplementary figures and images for: H4-methylation regulators mediated epitranscriptome patterns and tumor microenvironment infiltration characterization in hepatocellular carcinoma
Source: Clin Epigenetics. 2023 Mar 17;15:43. doi: 10.1186/s13148-023-01460-6 (PMC10024435; doi:10.1186/s13148-023-01460-6)

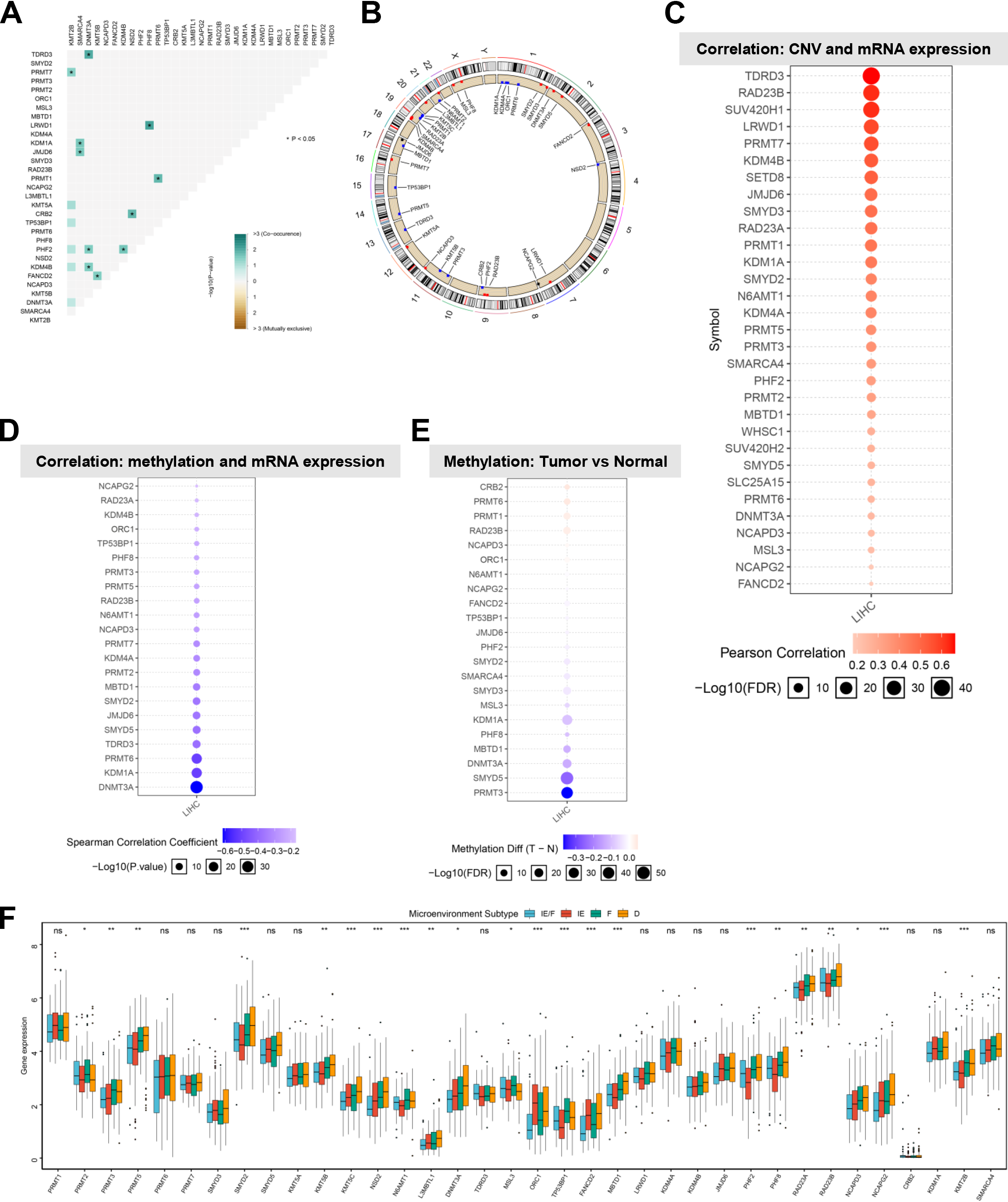

Supplement: Supplementary file 1 — Additional file 1. Figure S1: Correlation analyses for H4M regulators expression and CNV and methylation levels. A The mutation co-occurrence and exclusion analysis for histone methylation modification (H4M) regulators in the TCGA-LIHC cohort. Co-occurrence: aquamarine; exclusion: claybank. B The location of H4M modification genes on 23 chromosomes in TCGA-LIHC. C Correlation between mRNA expression and CNV variation levels of H4M regulators. D Correlation between mRNA expression and methylation levels of H4M regulators. E The differences in methylation level of H4M regulators between tumor and normal samples. F Expression of 36 H4M regulators between four microenvironment subtypes. IE/F: immune-enriched and fibrotic; IE: immune-enriched but non-fibrotic; F: fibrotic; D: immune-depleted. *, **, and *** mean p < 0.05, < 0.01, and < 0.001, respectively. [file 13148_2023_1460_MOESM1_ESM.tif]

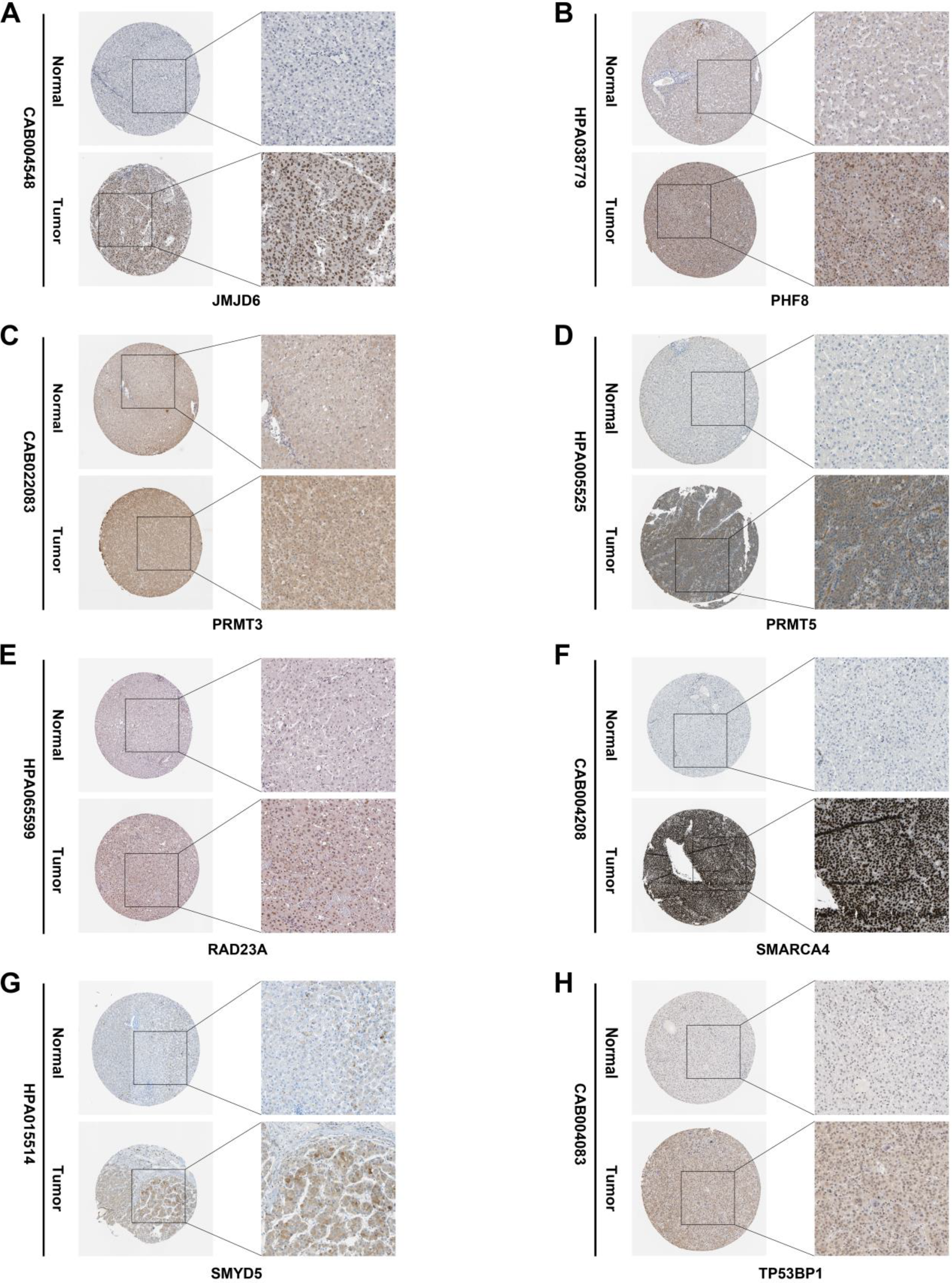

Supplement: Supplementary file 2 — Additional file 2. Figure S2：Immunohistochemistry of H4M regulators. A-H The protein levels of H4M regulators in normal liver and LIHC were visualized by immunohistochemistry in HPA. [file 13148_2023_1460_MOESM2_ESM.tif]

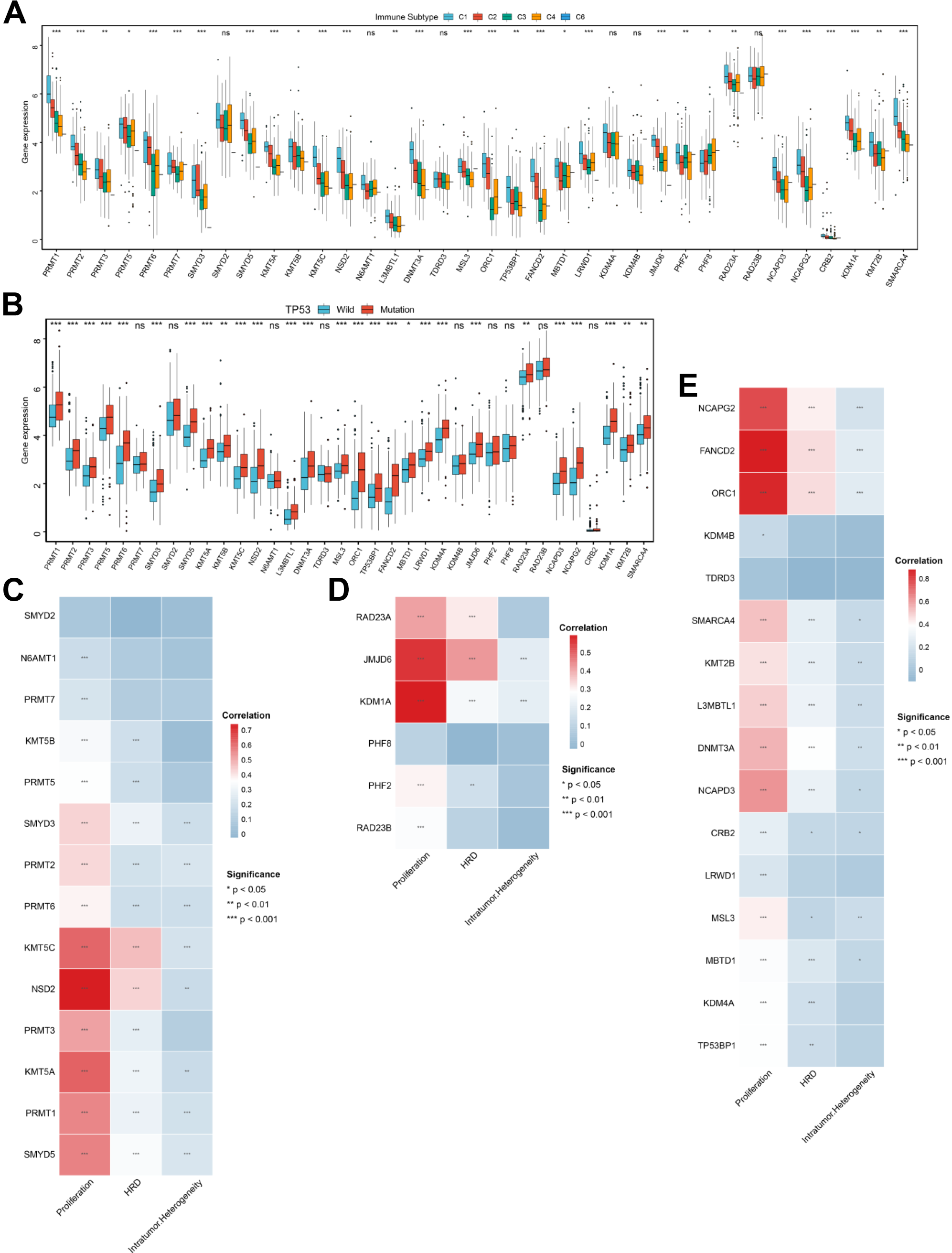

Supplement: Supplementary file 3 — Additional file 3. Figure S3: The expression landscape of H4M regulators in different subtypes and its correlation with different cancer markers. A Expression of 36 H4M regulators between six immune subtypes. B Expression of 36 H4M regulators between p53 wild and mutation groups. Three cancer signatures were involved, including homologous recombination deficiency (HRD), intratumor heterogeneity, and proliferation score. The correlation between these three cancer signatures and H4M writers was determined. C, H4M erasers D, and H4M readers E, respectively. *, **, and *** mean p < 0.05, < 0.01, and < 0.001, respectively. [file 13148_2023_1460_MOESM3_ESM.tif]

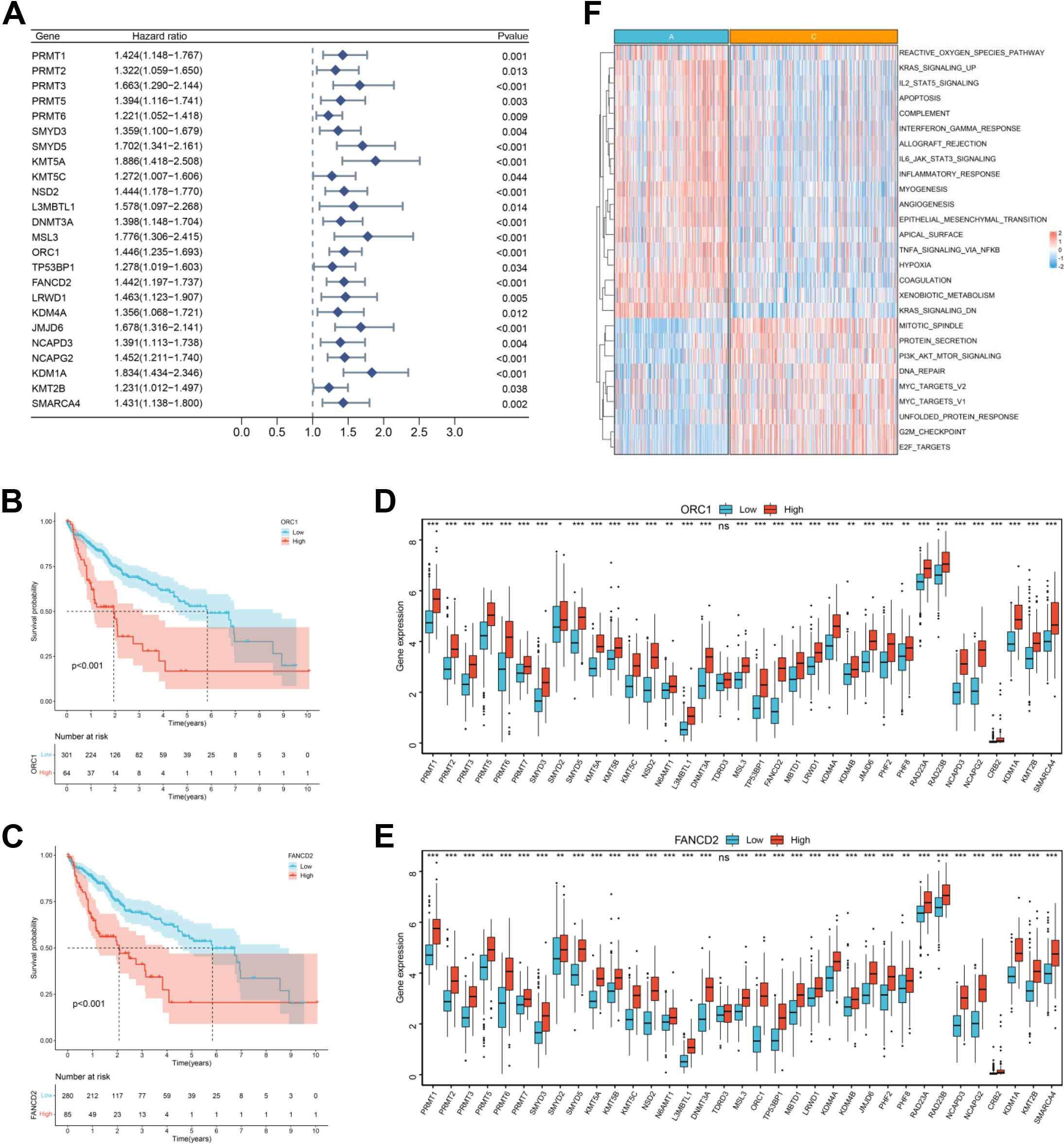

Supplement: Supplementary file 4 — Additional file 4. Figure S4: Prognostic analysis for H4M regulators and the importance of ORC1 and FANCD2 in overall H4M modification. A Expression of H4M regulators between ORC1 high expression group and low expression group. B Expression of H4M regulators between FANCD2 high expression group and low expression group. C The forest plot of the HR for the correlation between H4M regulators and the prognosis of HCC patients. D Kaplan–Meier survival analyses for the high and low ORC1 expression groups. E Kaplan–Meier survival analyses for the high and low FANCD2 expression groups. F The difference in enriched hallmarks between H4Mcluster-A and H4Mcluster-C. *, **, and *** mean p < 0.05, < 0.01, and < 0.001, respectively. [file 13148_2023_1460_MOESM4_ESM.tif]

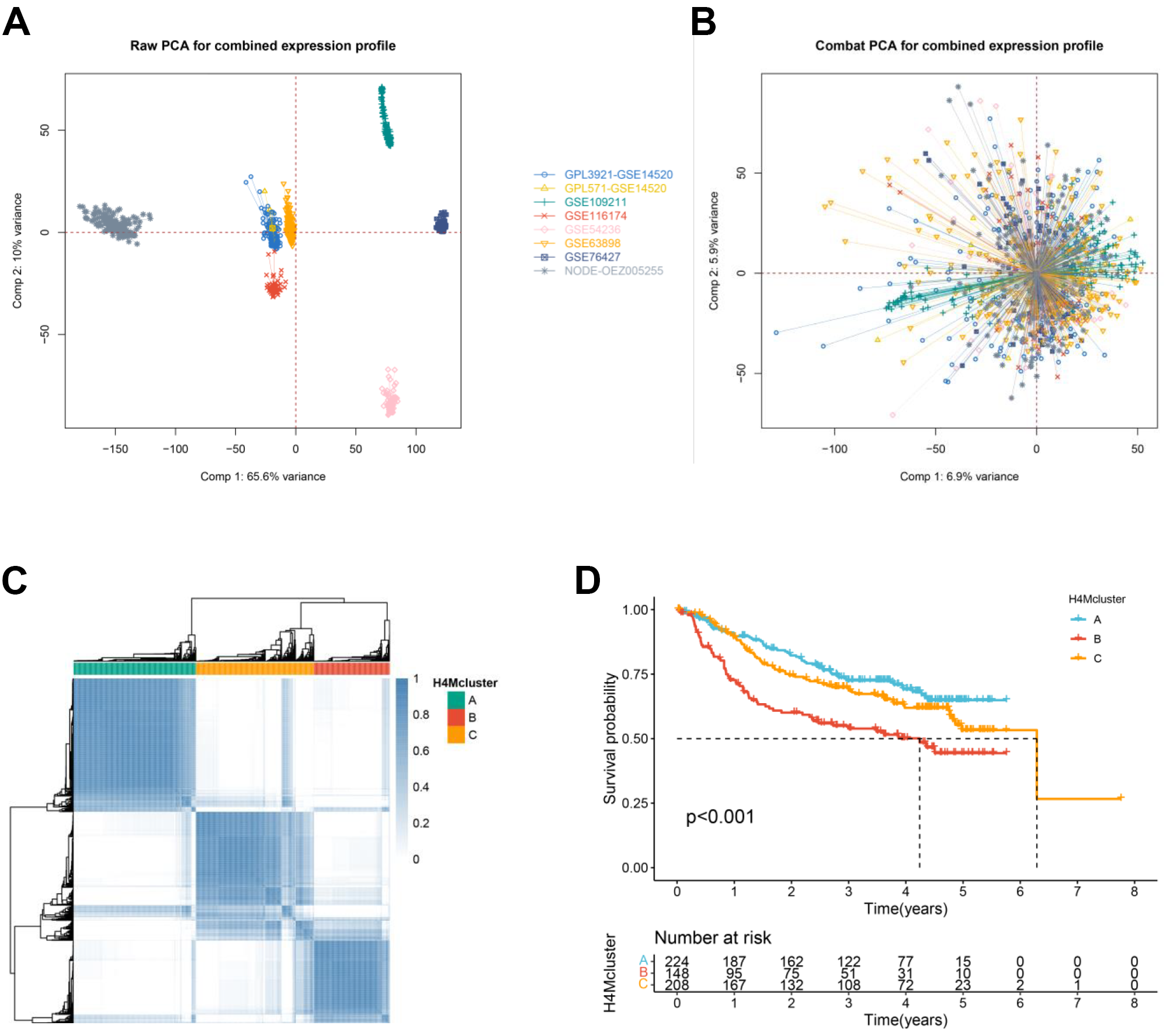

Supplement: Supplementary file 5 — Additional file 5. Figure S5: Three H4M modification patterns were validated in the integrated external cohort. A The distribution of eight data sets before consolidation. B The distribution of eight data sets after removing the batch effects. C Consensus matrix heatmap defining three H4Mclusters (k = 3) and their correlation areas. D Kaplan–Meier survival analysis for H4Mclusters. [file 13148_2023_1460_MOESM5_ESM.tif]

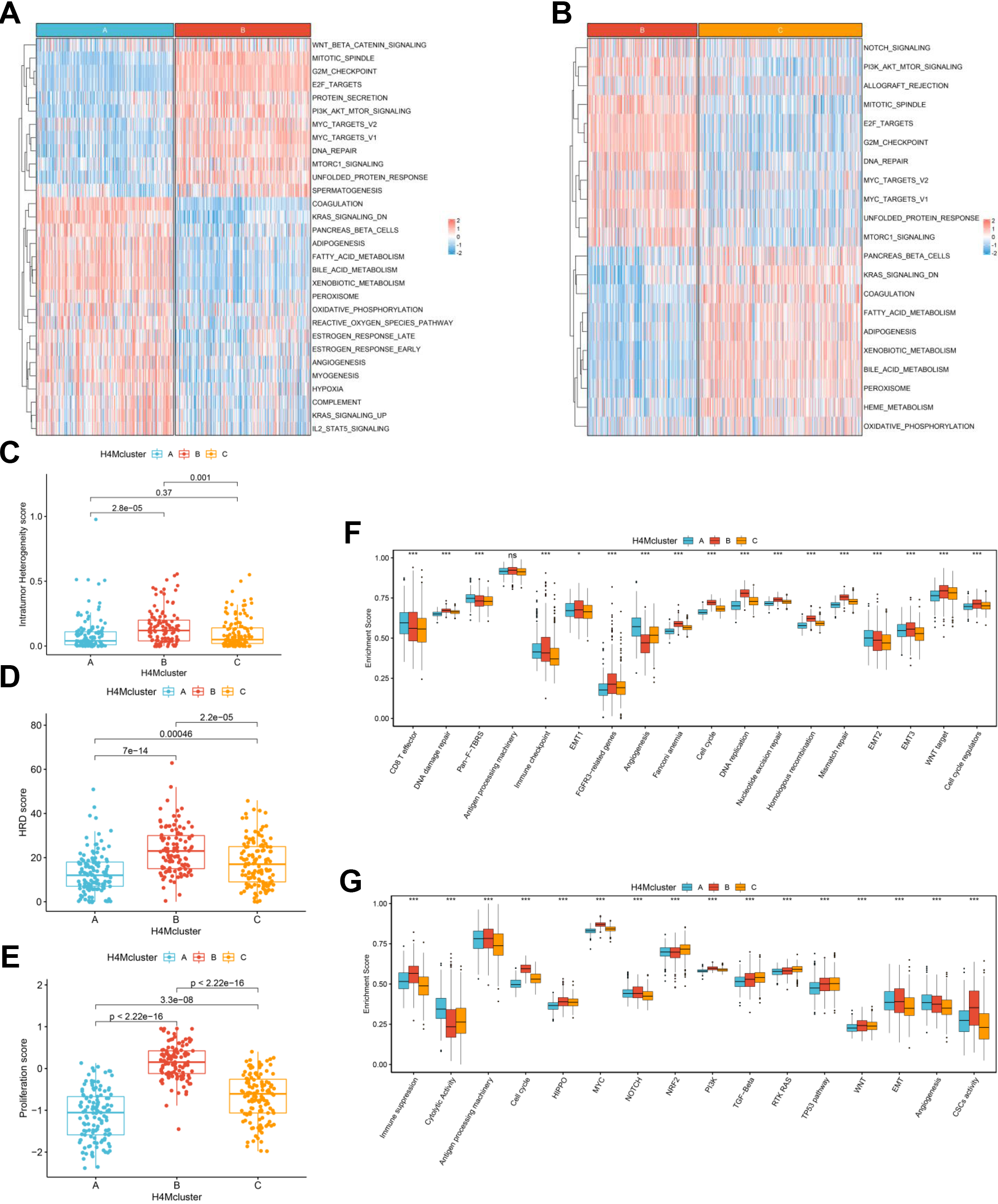

Supplement: Supplementary file 6 — Additional file 6. Figure S6: Biological function differences among three distinct H4M modification patterns. A Difference in enriched hallmarks between H4Mcluster-A and H4Mcluster-B. B Difference in enriched hallmarks between H4Mcluster-B and H4Mcluster-C. C Differences in intratumor heterogeneity score among three H4Mclusters. D Differences in HRD score among three H4Mclusters. E Differences in proliferation score among three H4Mclusters. F-G Two groups of typical cancer signatures differences among three H4Mclusters. *, **, and *** mean p < 0.05, < 0.01, and < 0.001, respectively. [file 13148_2023_1460_MOESM6_ESM.tif]

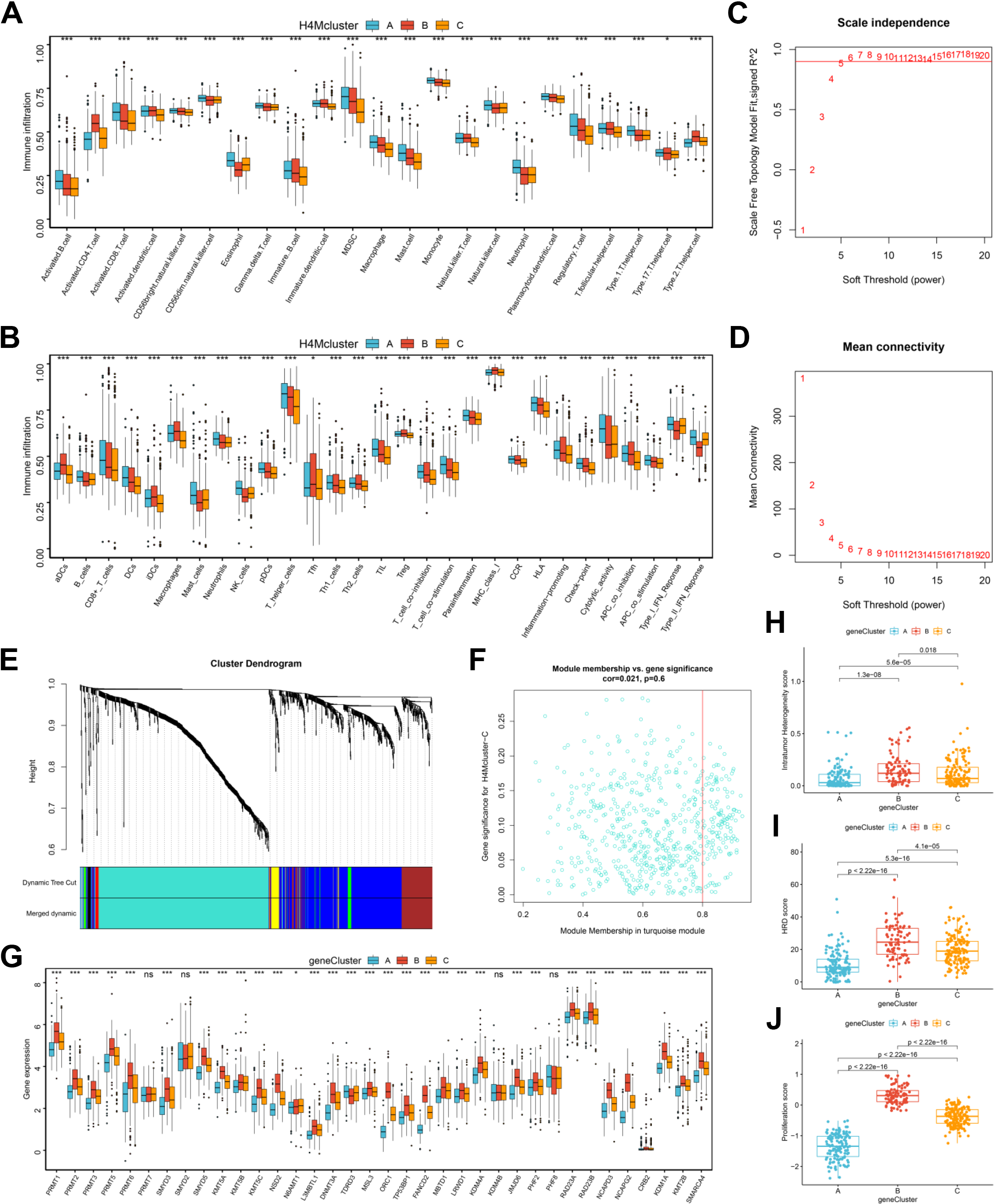

Supplement: Supplementary file 7 — Additional file 7. Figure S7: Immune infiltration evaluation and WGCNA analysis process. A-B Evaluating tumor-infiltrating immune cell abundance differences among three H4Mclusters. C-E The detailed analysis process of identifying key modules by WGCNA analysis. F Scatterplots of correlation between MEturquoise membership and gene significance for H4Mcluster-C. G Expression of 36 H4M regulators among three H4M geneClusters. H-J Differences in intratumor heterogeneity score, HRD score, and proliferation score among three H4M geneClusters. *, **, and *** mean p < 0.05, < 0.01, and < 0.001, respectively. [file 13148_2023_1460_MOESM7_ESM.tif]

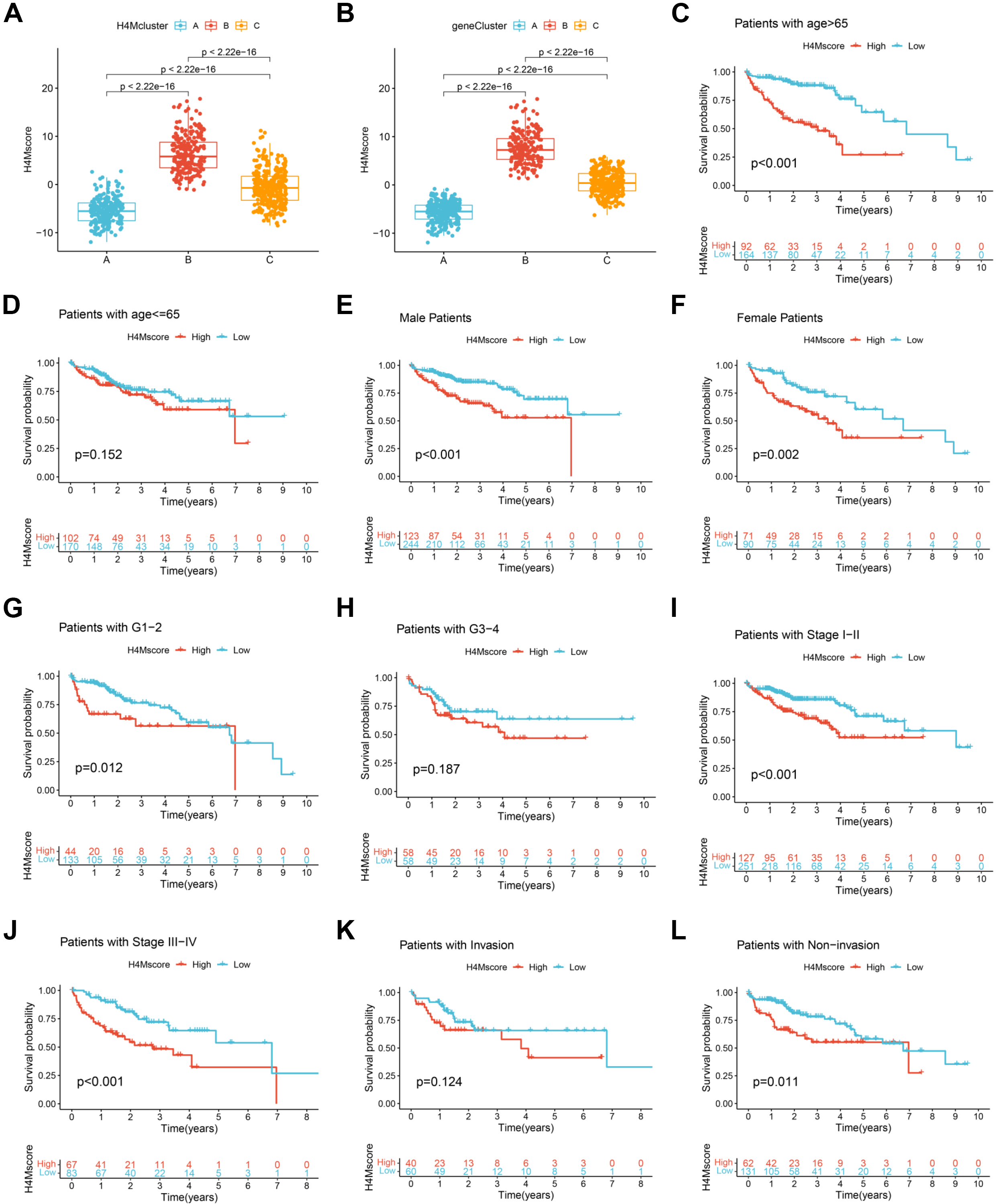

Supplement: Supplementary file 8 — Additional file 8. Figure S8: Validating the prognostic value of H4Mscore in the internal and external cohorts. A-C Kaplan–Meier survival analyses for the high and low H4Mscore groups in three internal cohorts, including TCGA, ICGC-LIRI, and ICGA-LICA, respectively. D-H Kaplan–Meier survival analyses for the high and low H4Mscore groups in five external cohorts, including NODE-OEZ005255, GPL3921-GSE14520, GSE76427, GSE116174, and GPL571-GSE14520, respectively. [file 13148_2023_1460_MOESM8_ESM.tif]

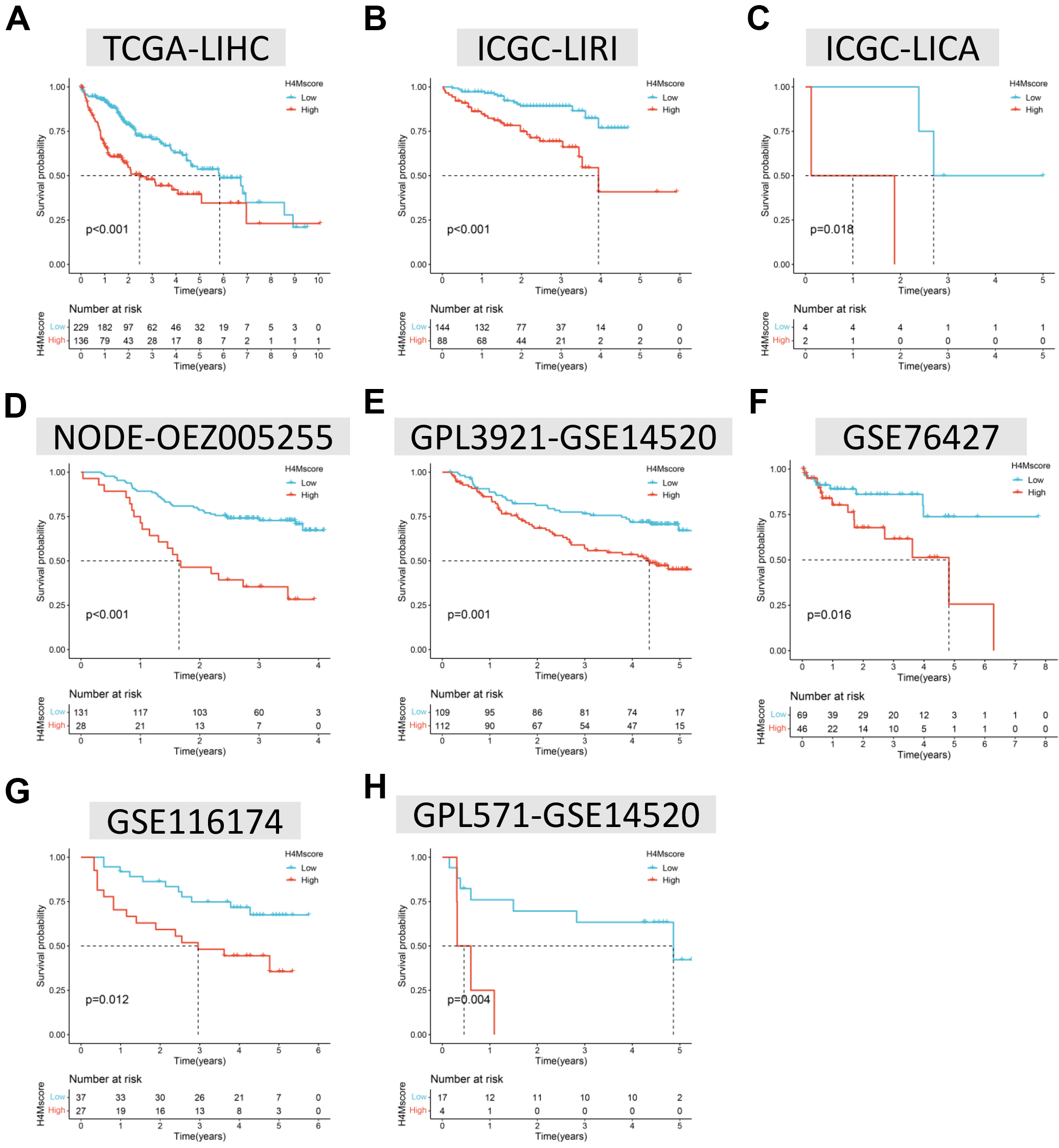

Supplement: Supplementary file 9 — Additional file 9. Figure S9: Validating the prognostic value of H4Mscore in different clinical subtypes. A-B Difference in H4Mscore among three H4Mclusters and three H4M geneClusters, respectively. C-L Kaplan–Meier survival analyses for the high and low H4Mscore groups in different subtypes, including age, gender, histologic grade, TNM stage, and vascular invasion, respectively. [file 13148_2023_1460_MOESM9_ESM.tif]

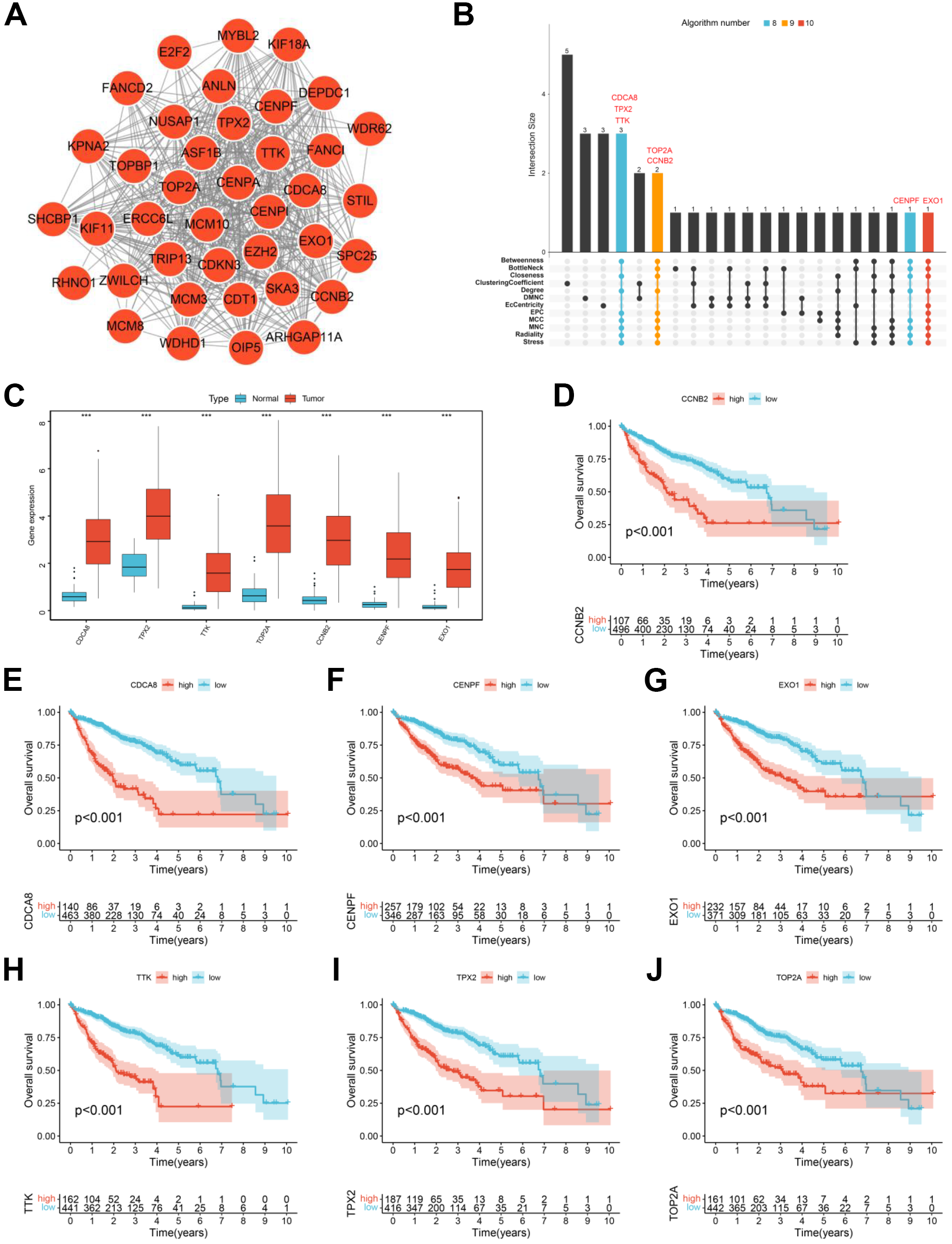

Supplement: Supplementary file 10 — Additional file 10. Figure S10: Selection of hub genes for further experiments and validation. A The protein–protein interaction network. B The hub genes are selected by 12 cytoHubba algorithms in the Cytoscape software. C Expression of seven core genes between normal and HCC samples. D-J The Kaplan–Meier survival analyses for seven filtered hub genes. *, **, and *** mean p < 0.05, < 0.01, and < 0.001, respectively. [file 13148_2023_1460_MOESM10_ESM.tif]

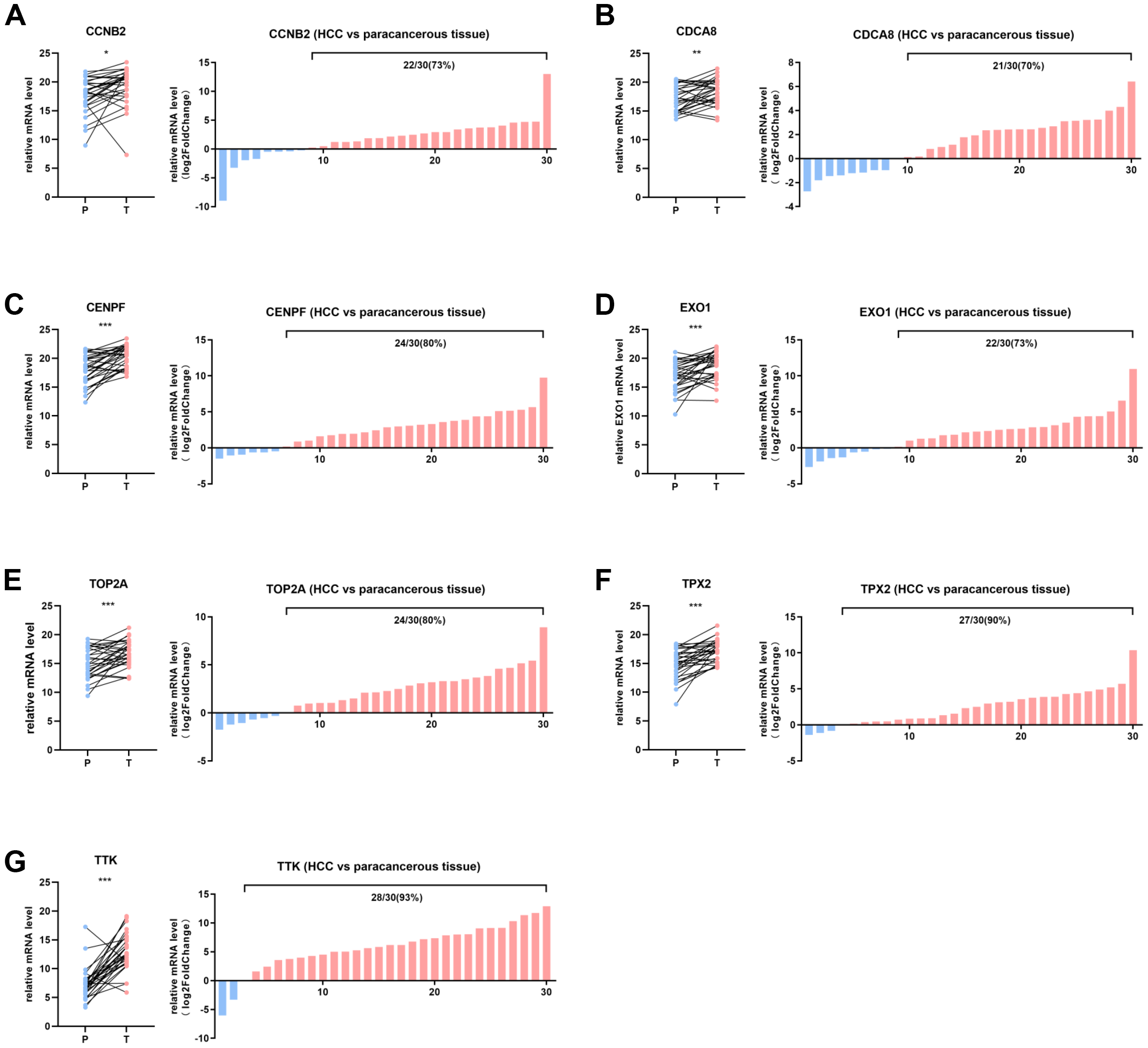

Supplement: Supplementary file 11 — Additional file 11. Figure S11: Expression of seven core genes in clinical hepatocellular carcinoma (HCC) samples and paired paracancerous samples. A-G mRNA expression of seven core genes in HCC samples (T) and paracancerous samples (P). *, **, and *** mean p < 0.05, < 0.01, and < 0.001, respectively. [file 13148_2023_1460_MOESM11_ESM.tif]

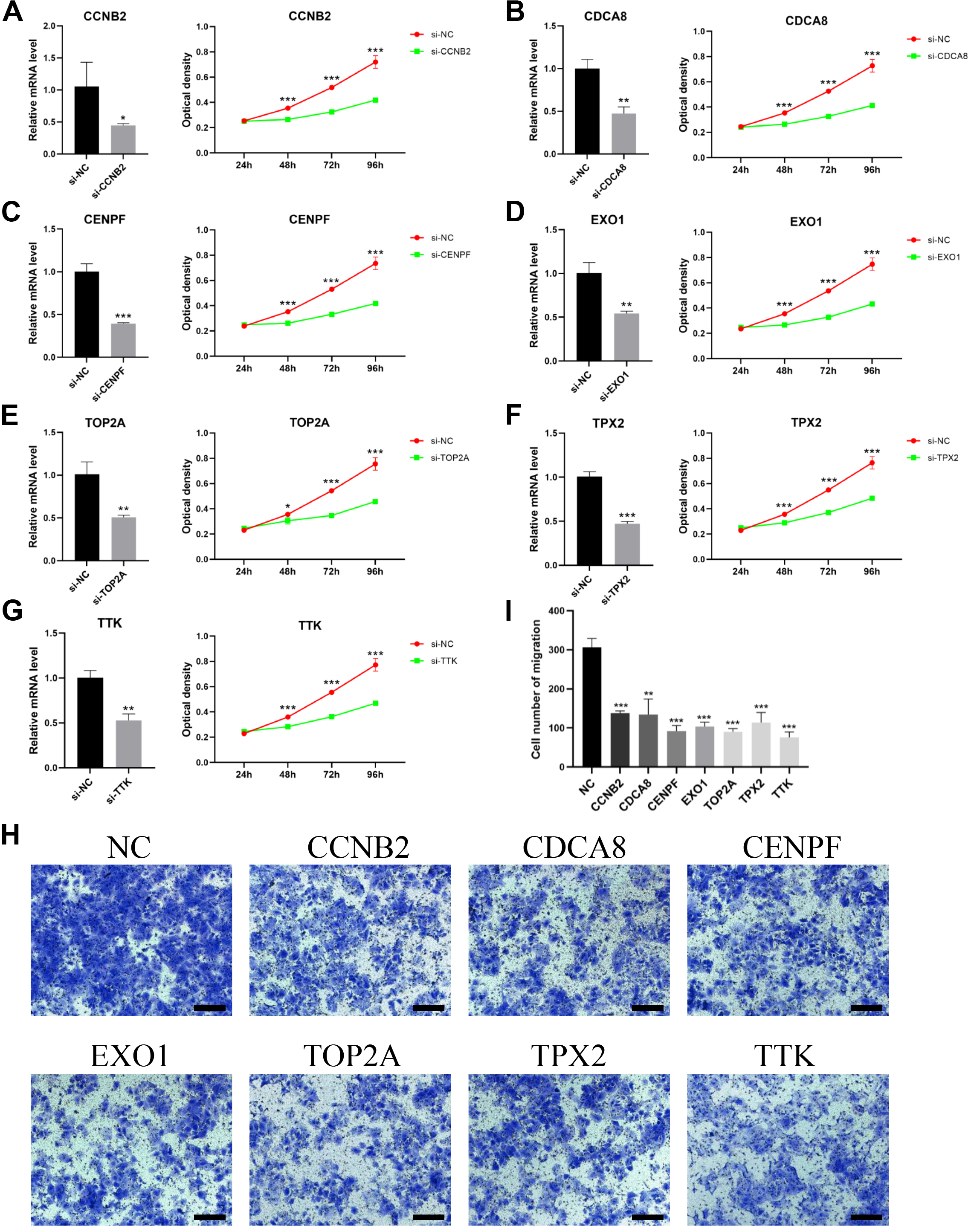

Supplement: Supplementary file 12 — Additional file 12. Figure S12: Effects of hub genes on the proliferation and migration of HCC cells. After being treated with siRNAs, the mRNA expression and optical density curves of CCNB2 (A), CDCA8 (B), CENPF (C), EXO1 (D), TOP2A (E), TPX2 (F), and TTK (G) in Huh‐7 cells. H-I Migration experiment of Huh‐7 cells treated with siRNA for 48 hours. Cells were stained with crystal violet. Scale bar, 100 μM. *, **, and *** mean p < 0.05, < 0.01, and < 0.001, compared with negative control (NC) group. [file 13148_2023_1460_MOESM12_ESM.tif]

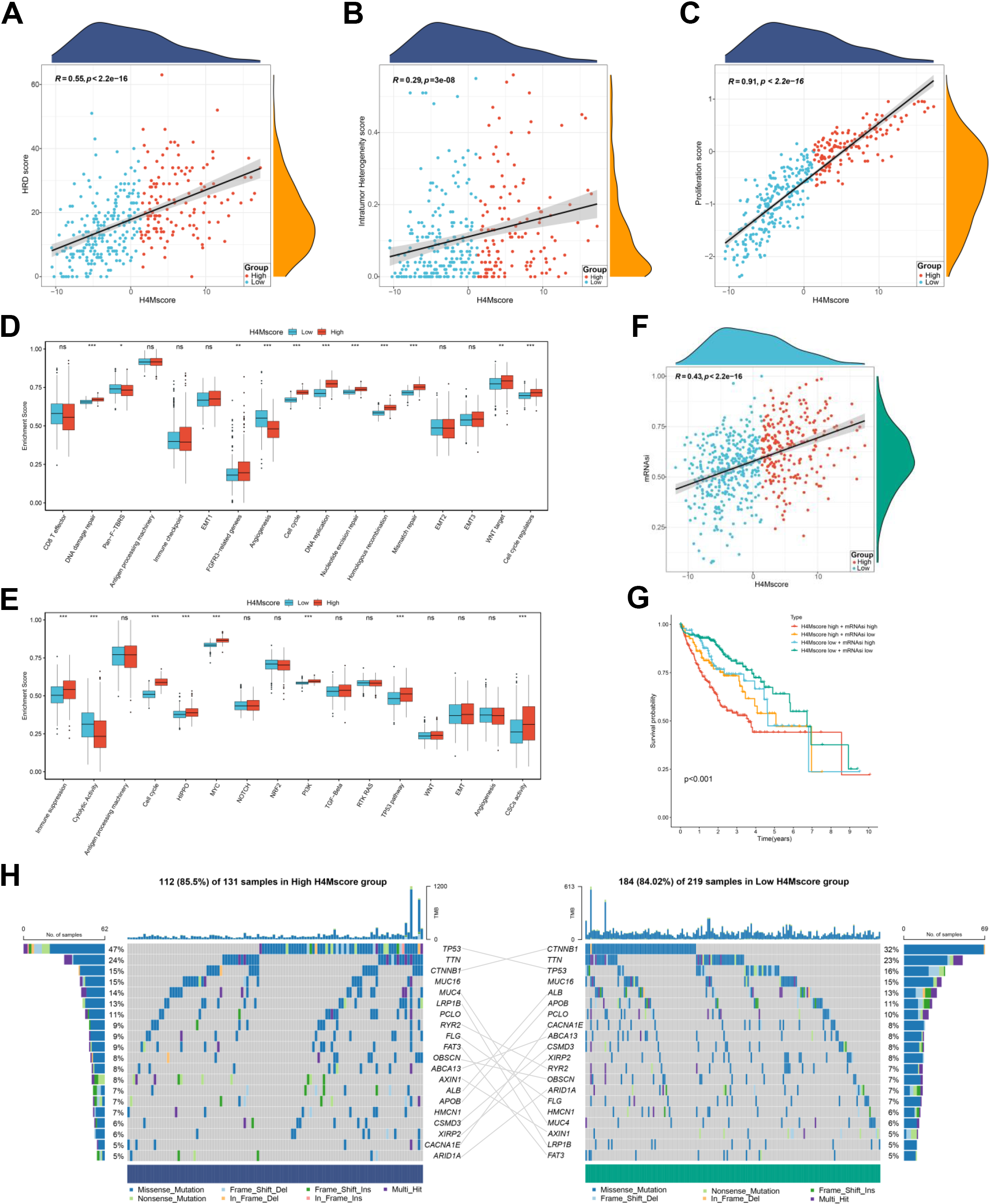

Supplement: Supplementary file 13 — Additional file 13. Figure S13: Correlation between H4Mscore and cancer signatures. By employing the Spearman method, correlation analyses between H4Mscore and HRD score (A), intratumor heterogeneity score (B), and proliferation score (C) were performed. (D-E) Two groups of typical cancer signatures differences between the high H4Mscore and low H4Mscore groups. (F) Correlation analyses between H4Mscore and mRNA stemness index (mRNAsi) using Spearman method. (G) Survival analyses for HCC patients stratified by both H4Mscore and mRNAsi using Kaplan–Meier curves. (H) The waterfall plot of tumor somatic mutation established by those with high H4Mscore and low H4Mscore. *, **, and *** mean p < 0.05, < 0.01, and < 0.001, respectively. [file 13148_2023_1460_MOESM13_ESM.tif]
